# Assessing spatial sequencing and imaging approaches to capture the molecular and pathological heterogeneity of archived cancer tissues

**DOI:** 10.1002/path.6383

**Published:** 2025-01-23

**Authors:** Tuan Vo, P Prakrithi, Kahli Jones, Sohye Yoon, Pui Yeng Lam, Yung‐Ching Kao, Ning Ma, Samuel X Tan, Xinnan Jin, Chenhao Zhou, Joanna Crawford, Shaun Walters, Ishaan Gupta, Peter H Soyer, Kiarash Khosrotehrani, Mitchell S Stark, Quan Nguyen

**Affiliations:** ^1^ The Institute for Molecular Bioscience The University of Queensland St Lucia Queensland Australia; ^2^ Queensland Institute of Medical Research Herston Queensland Australia; ^3^ School of Biomedical Sciences and Pharmacy University of Newcastle Callaghan New South Wales Australia; ^4^ Precision Medicine Research Program Hunter Medical Research Institute New Lambton Heights New South Wales Australia; ^5^ University of Queensland – IIT Delhi Research Academy (UQIDRA) New Delhi India; ^6^ Department of Biochemical Engineering and Biotechnology Indian Institute of Technology Delhi New Delhi India; ^7^ Genome Innovation Hub The University of Queensland St Lucia Queensland Australia; ^8^ Dermatology Research Centre The Frazer Institute, The University of Queensland Woolloongabba Queensland Australia; ^9^ Akoya Biosciences Inc Marlborough Massachusetts USA; ^10^ School of Biomedical Sciences The University of Queensland St Lucia Queensland Australia

**Keywords:** dysplastic naevus, melanoma, spatial transcriptomics, pathological annotation, formalin‐fixed paraffin‐embedded, poly(A)‐capture, probe‐capture, CODEX, RNAScope

## Abstract

Spatial transcriptomics (ST) offers enormous potential to decipher the biological and pathological heterogeneity in precious archival cancer tissues. Traditionally, these tissues have rarely been used and only examined at a low throughput, most commonly by histopathological staining. ST adds thousands of times as many molecular features to histopathological images, but critical technical issues and limitations require more assessment of how ST performs on fixed archival tissues. In this work, we addressed this in a cancer‐heterogeneity pipeline, starting with an exploration of the whole transcriptome by two sequencing‐based ST protocols capable of measuring coding and non‐coding RNAs. We optimised the two protocols to work with challenging formalin‐fixed paraffin‐embedded (FFPE) tissues, derived from skin. We then assessed alternative imaging methods, including multiplex RNAScope single‐molecule imaging and multiplex protein imaging (CODEX). We evaluated the methods’ performance for tissues stored from 4 to 14 years ago, covering a range of RNA qualities, allowing us to assess variation. In addition to technical performance metrics, we determined the ability of these methods to quantify tumour heterogeneity. We integrated gene expression profiles with pathological information, charting a new molecular landscape on the pathologically defined tissue regions. Together, this work provides important and comprehensive experimental technical perspectives to consider the applications of ST in deciphering the cancer heterogeneity in archived tissues. © 2025 The Author(s). *The Journal of Pathology* published by John Wiley & Sons Ltd on behalf of The Pathological Society of Great Britain and Ireland.

## Introduction

As cancer is a genetically heterogeneous disease, multimodal and multiplex molecular data are increasingly being used to aid cancer diagnosis, prognosis, and treatment decisions [1]. Spatial transcriptomics (ST) applications for fresh‐frozen specimens have characterised tumour heterogeneity at an unprecedented resolution [2, 3]; however, this embedding type is often not available for a large‐scale, long‐term study with clinical follow‐ups. Formalin‐fixed paraffin‐embedded (FFPE) tissues present a vast archival biological resource and are used in all routine histopathology diagnostic laboratories [4]. Despite the many advantages of these abundant and precious clinical FFPE samples, they remain underutilised for transcriptomic profiling due to formaldehyde cross‐linking and perceived RNA degradation, which can impair the performance of conventional ST pipelines [5, 6, 7, 8]. Recently, several spatial platforms, such as Visium FFPE or Visium CytAssist (10x Genomics, Pleasanton, CA, USA) [9], CosMX Single Molecular Imager (SMI, NanoString, Seattle, WA, USA) [10], Imaging Mass Cytometry (e.g. Hyperion, Fluidigm) [11], and Co‐detection By Indexing (CODEX/PhenoCycler, Akoya Biosciences, Marlborough, MA, USA) [12], have been developed to capture and quantify RNA and protein from FFPE tissues. However, these methods depend on predesigned probe panels, which are limited in many use cases. Here we assessed a range of new experimental and analytical methods available for exploring FFPE tissues, including our own customisation protocols, and with an emphasis on methods allowing target discovery beyond that of known gene panels.

Clinical diagnostic practice remains driven by haematoxylin‐eosin (H&E) images, which typically rely on qualitative and highly variable manual interpretation. Breakthroughs assisting pathologists in using the rich information in cancer biopsies are required to increase the precision of clinical decisions and to advance the systemic and mechanistic understanding of cancer. Unlike traditional genomics technologies such as bulk and single‐cell RNA sequencing, which require tissue dissociation, ST allows for the full retention of a tumour's spatial and anatomical context [13]. The Visium FFPE ST platform (10x Genomics) is a technology capable of measuring ~18,000 genes representing the whole transcriptome while generating histological‐grade H&E images, allowing for pathological annotation. Fluorescence *in situ* hybridisation (FISH) methods or other spatially resolved multiplex protein detection methods, such as imaging mass cytometry and CODEX/PhenoCycler, currently provide single‐cell spatial resolution; however, these are limited to a small number of markers at present.

In this study, we assessed a fresh frozen poly(A)‐capture Visium protocol modified for FFPE samples (hereafter referred to as ‘poly(A)‐capture protocol’) and the probe‐based protocols of Visium ST (‘probe‐capture protocol’). As the polyA‐capture protocol has the potential to detect known and new long non‐coding RNAs in ST data, we applied a computational method to map long non‐coding RNAs (lncRNAs) from spatial tissue. We aimed to develop a FFPE ST workflow allowing for deep interrogation of the transcriptional complexity and morphological characteristics of this challenging pathology without the need for the use of limited fresh tissue samples. We adopted, compared, and combined the two ST platforms for archived human FFPE tissue. One of our key contributions was the thorough assessment of the pipeline across a broad range of tissue quality and storage times.

Both protocols were assessed using human FFPE melanoma and dysplastic naevi tissues (atypical moles). Melanoma, an aggressive and heterogeneous skin cancer [14, 15], has been analysed using various methods, including gene expression profiling [16, 17], immunohistochemistry (IHC) [18], proteomic assays [19, 20], and fresh frozen spatial transcriptomics [21]; however, the results were still limited by the absence of spatial context and low coverage of the transcriptome or proteome. Notably, skin biopsies represent the most challenging samples to obtain a consistent high‐quality transcriptome [22], especially for old and low‐quality archival FFPE tissues, mainly due to the fibrous tissue composition and possible RNAse contamination. Through the integration of histopathological annotation with spatial sequencing data, we quantified a high level of tumour heterogeneity. Further, we assessed imaging‐based methods, where the detection of cell types and gene markers from ST data was compared with the highly sensitive, single‐cell‐resolution RNAScope imaging method and the CODEX/PhenoCycler, multiplex protein imaging assay. Overall, we comprehensively assessed the FFPE ST pipeline, suggesting a high potential for an integrated approach for utilising valuable archival tissues for investigating biological insights and opening a huge potential for clinical applications.

## Materials and methods

### FFPE samples and RNA quality control

Included in this study were clinical FFPE biopsies of dysplastic naevi and melanoma, of various archival ages, RNA quality, and patient disease stages (supplementary material, Table S1). Institutional approval of experiments involving human tissues was provided by Metro South and The University of Queensland Human Research Ethics Committees HREC/17/QPAH/817, 2018000165, and 2017000318.

FFPE blocks were previously prepared in a standard procedure with fixation in 10% formalin, processed in ethanol and xylene, and embedded in paraffin wax. All blocks were stored at room temperature. To assess the suitability of each sample for transcriptomic analysis, 7‐μm sections were collected in triplicate per sample for RNA extraction using an RNeasy FFPE Kit (Catalogue No.: 73504, Qiagen, Hilden, Nordrhein, Germany). The RNA Integrity Number (RIN) and DV200 were determined by BioAnalyzer electrophoresis using an RNA 6000 Pico Kit (Catalogue No.: 5067‐1,513, Agilent, Santa Clara, CA, USA). The DV200 metric refers to the percentage of total profiled RNA fragments greater than 200 bp in length, with scores of at least 30% considered acceptable for sequencing applications [23, 24]. An increasing number of fragments below this threshold in a sample is indicative of an increasing degree of RNA degradation. For this project, we selected samples with a large range of DV200 scores (supplementary material, Figures S1 and S2), aiming to assess the effect of FFPE RNA degradation on spatial transcriptomic data quality.

### Poly(A)‐capture

**Figure 1:**
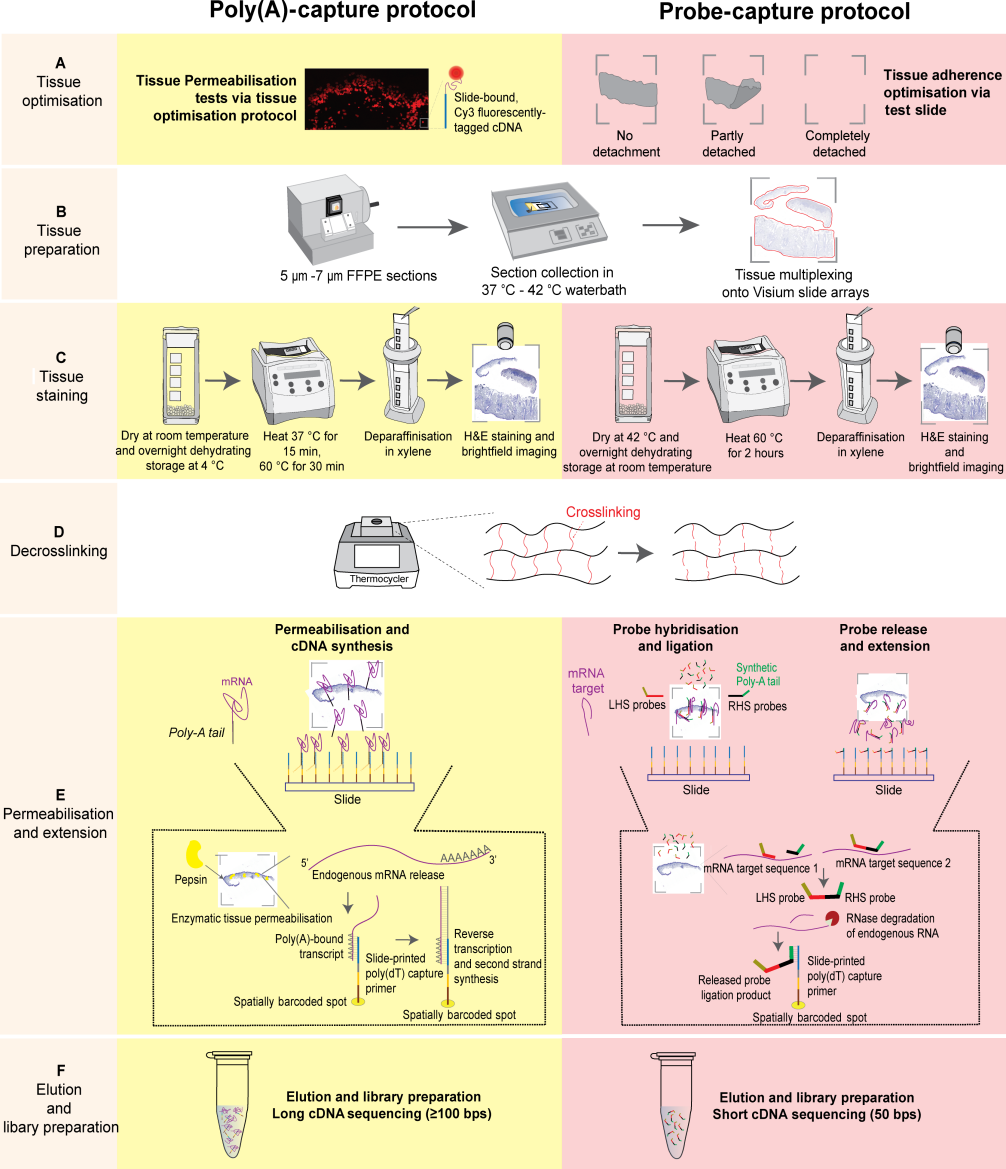
Developing and assessing protocols to perform spatial transcriptomics to capture thousands of genes in FFPE cancer tissue. (A) Poly(A)‐capture required the optimisation of tissue permeabilisation step. Probe‐capture required a tissue adherence test. (B) Optimisation for tissue multiplexing and sectioning thickness. (C) Tissue stainning was processed in different conditions in two protocols. (D) Decrosslinking was performed in the same way. (E) In permeabilisation, the mRNA molecules or hybridised probes were released from cells and bound to the spatial oligos on the glass slide. Reverse transcription produced cDNA products in poly(A)‐capture protocol or extended probes in probe‐capture protocol. (F) Eluting captured molecules/probes and preparing the library for long/short cDNA sequencing.

We have further optimised a recent protocol [25], improving tissue handling and adherence for FFPE melanoma and dysplastic naevi samples (detailed in Figure 1).

#### Tissue optimisation

The FFPE tissue sections were collected at 7‐μm thickness, trimmed to include pathologist‐annotated regions of interest (ROIs) (i.e. melanoma, stromal, and immune cell infiltrated regions), and positioned on a Visium Tissue Optimisation slide (Catalogue No.: 3000394). The slides were then dehydrated, stored overnight, dried, and deparaffinised by heat (60 °C) and xylene (5 min, twice). Tissues were then rehydrated using an ethanol gradient (100% for 2 min, twice; 90% for 2 min, twice; 85% for 2 min) before being stained with H&E and imaged using a Zeiss AxioScan Z1 slide scanner (Jena, Thuringen, Germany). Next, decrosslinking was performed by incubation in collagenase (Catalogue No.: 17018‐029) and then 1X Tris‐EDTA buffer (pH 8.0). Tissue sections were then immediately permeabilised using pepsin 0.1% (LS003319) in an increasing incubation time series (5–40 min). Finally, cDNA was synthesised from the captured RNA, fluorescently labelled with cyanine 3 (Cy3), and visualised using a Leica DMi8 inverted widefield microscope (Leica Microsystems, Wetzlar, Hesse, Germany).

#### Visium spatial gene expression library preparation for skin cancer tissues

Following optimisation of the aforementioned conditions, FFPE blocks were sectioned and placed on Visium Spatial Gene Expression Slides (Catalogue No.: 2000233). Tissues were permeabilised for the optimised period of time determined through experimental testing using the Visium Tissue Optimisation slide (25 min) as described in the previous step. cDNA was synthesised *in situ* from slide‐bound poly(A) RNA, followed by second‐strand synthesis and denaturation. The denatured, full‐length cDNA strands were PCR amplified for 19 or 20 cycles. Amplified cDNA was end‐repaired, A‐tailed, and size‐selected using SPRIselect (0.8X bead cleanup). Illumina TruSeq Read 2 sequences were ligated and standard i5 and i7 sample indexes added.

All libraries were loaded at 1.8 pm onto a NextSeq500 High Output 150‐cycle kit (Illumina, San Diego, CA, USA). For the probe‐capture protocol, a minimum of 25,000 read pairs per spot is required, and the sequencing cycles used were as follows: Read 1 at 28 cycles, i7 and i5 indices at 10 cycles each, and Read 2 at 50 cycles. For the polyA‐capture protocol, a minimum of 50,000 read pairs per spot is desirable, and we used the same set up with Read 1 at 28 cycles, i7 and i5 indices at 10 cycles each, and Read 2 at 120 cycles.

### Probe‐capture

The probe‐capture protocol was based on the Visium Spatial Gene Expression for FFPE User Guide (CG000407, CG000408, CG000409 – 10x Genomics), with modifications as optimised for melanoma and naevus tissue.

#### Tissue adherence optimisation

FFPE tissues were collected at 5‐μm thickness and trimmed to include pathologist‐annotated ROIs, then multiplexed onto the Visium Tissue Section Test Slides (Catalogue No.: 2000460). The slides were later dried, stored overnight, and deparaffinised by heat and xylene. The tissues were rehydrated by an ethanol gradient following 10X protocol (CG000409), followed by H&E staining and imaging. Finally, decrosslinking was carried out using 1X TE buffer (pH 8.0) for 1 h at 70 °C (with preconditioning in HCl).

#### Library preparation

To prepare probe‐capture libraries for sequencing, FFPE sections were multiplexed onto Visium Spatial Gene Expression Slides. The process followed 10X user guide (CG000407, CG000409), utilising the whole transcriptome (18,000 protein coding genes) human probe set (Catalogue Nos.: 2000449 and 2000450). Sequencing was performed using NextSeq500 (Illumina).

### Multiplexed RNA *in situ* hybridisation using RNAScope assays

Based on known skin‐cancer‐associated markers, we selected six gene targets for probe design by ACDBio utilising RNAScope HiPlex12 Reagent Kit (Advanced Cell Diagnostics, Inc., Newark, CA, USA) version 2 standard assay (ACD Catalogue No.: 32442): CTLA4 (ADV554341‐T6), SOX10 (ADV484121‐T7), Keratin8, 18 & 19 (ADV404751‐T8), CD8 (ADV560391‐T9), Ki67 (ADV548881‐T11), and CD4 (ADV605601‐T12). The assay was performed following the manufacturer's user manual (UM 324409). In brief, FFPE melanoma tissues were sectioned at 5 μm, collected onto slides, and then dried at 60 °C for 2 h prior to deparaffinisation. Subsequently, the target retrieval step was performed followed by protein digestion with Protease III (Catalogue No.: 322340). The slide was then incubated with a mixture of the six test probes or control probes for hybridisation with RNA. After signal amplification, the slides were incubated with RNAScope Hiplex FFPE reagent to reduce the autofluorescence of the FFPE tissues. The tissue was counterstained with DAPI and mounted under a coverslip. Imaging was performed using a Zeiss LSM900 with a 63× oil objective and five filters (DAPI, FITC, Cy3, Cy5, and Cy7). Between imaging rounds, coverslips were removed, and fluorophores of previous imaging rounds were cleaved to enable consecutive rounds of imaging, with each round containing probes for a new set of transcripts. The single‐channel image at each round of imaging was saved and used to generate the composite image using RNAScope HiPlex Image Registration Software version 2.0.1.

### Data analysis

Sequencing data were mapped and demultiplexed (10x SpaceRanger) and subsequently analysed through our software package, stLearn [26]. The analysis consisted of (1) mapping raw fastq data and calculating read counts per gene per spot, (2) overlaying gene expression data with H&E stained tissue images, (3) performing normalisation using stSME [26] and integration followed by unsupervised Louvain clustering, (4) differential expression analysis between spatial clusters Wilcoxon rank‐sum test [26], and (5) visualisation of gene markers for clusters. Heterogeneity was then assessed at two levels, genes and cell types. To discriminate cell types, ST‐derived clusters were assigned functional names by gene markers, representing dominant cell types or tissue organisation. (6) We used robust cell‐type decomposition (RCTD) [27] to annotate the cell types by using a scRNA‐seq reference [28]. Cell‐type plots were generated with stLearn [29].

### Non‐coding RNA detection from spatial data

The ST data from both pipelines were compared for their ability to capture long non‐coding RNAs. A computational algorithm [30] was adopted to identify transcriptionally active regions. Briefly, the pipeline uses an R package groHMM [31] that utilises a two‐state hidden Markov model to classify regions in an aligned genome as transcriptionally active or not, based on the read coverage in each bin. The position‐sorted BAM files generated by the 10X Space Ranger pipeline were used as inputs to the pipeline. By default, it splits the genome into non‐overlapping bins of 50 bp, and a bin is called transcriptionally active if reads are detected in that bin and are labelled transcriptionally active regions (TARs). TARs found no more than 500 bp apart are merged into one unit. The regions identified are then overlapped with reference gene annotations (reference annotations from 10X). TARs overlapping with existing gene annotations are labelled aTARs (annotated TARs) and those falling outside gene boundaries are called uTARs (unannotated TARs). We overlapped these with existing databases for lncRNAs like FANTOM [32] and LncExpDB [33] in a strand‐specific manner to identify previously reported lncRNAs. We identified lncRNAs significantly differentially expressed in melanocytes and T cells using a non‐parametric test in Seurat [34]. The spatial expression of cell‐type‐specific lncRNAs and of corresponding cell‐type marker genes [16] were visualised on the tissue with stLearn [29] to compare the spatial distribution patterns.

### Phenocycler‐fusion (Akoya Biosciences)

Single‐cell spatial phenotyping of the melanoma FFPE slides was performed in collaboration with Akoya Biosciences (The Spatial Biology Company, Marlborough, MA, USA) on the Phenocycler®‐Fusion (PCF) platform. The tissue slides were stained with a 38‐plex antibody panel for various markers (supplementary material, Table S2). PCF immunostaining was performed according to the PCF user manual and previously published method from Goltsev, *et al* [35] and Black, *et al* [36]. This process involved cycles of reporter hybridisation, imaging, and dehybridisation to capture images. The images from all cycles were compiled into a final composite image, which was analysed using QuPath software [37] to reveal the spatial expression patterns of the markers.

## Results

### Optimisation of spatial transcriptomics protocols for FFPE skin samples

Archived FFPE melanoma and dysplastic naevus tissues pose challenges in measurement in an unbiased and untargeted way. Here we bridge this disparity through the successful optimisation of two alternate sequencing‐based ST protocols for these tissues (Figure 1). In the poly(A)‐capture protocol, we optimised the sectioning, deparaffinisation, decrosslinking, and permeabilisation conditions. Here, optimisation of sectioning thickness, deparaffinisation, decrosslinking, and permeabilisation is important for tissue attachment and the optimal release of RNA to be captured by oligo‐dT probes on the spatial slides. Over‐permeabilisation will lead to diffusion and non‐specific signals, while underpermeabilisation will cause incomplete release of RNA and loss of signals. We also found that the standard Visium Spatial Gene Expression manufacturer's protocol required further optimisation for FFPE skin samples (probe‐capture protocol). The probe‐capture protocol uses RNA‐templated hybridisation probes and is expected to ensure high sensitivity and specificity that could be compromised for poly(A)‐capture, which relies solely on long poly(A) tail sequences. Figure 1 presents a step‐by‐step comparison of these two optimised protocols.

A primary point of optimisation, commonly required for FFPE samples, is to maximise tissue adherence to the ST slide. Initially, we observed tissue detachment in both melanoma and naevus samples throughout deparaffinisation, staining, and decrosslinking, particularly for small, overly dehydrated, and fragile tissues with a high propensity for detachment. For the poly(A) workflow, we performed custom optimisations prior to running the default tissue optimisation slides. Improved adherence was observed after rehydrating FFPE blocks in cold water prior to sectioning, decreasing section thickness to 7 μm, drying the slide before storing overnight with desiccator beads, and increasing the wax‐melting temperature. Comparatively, for the probe‐capture workflow, a tissue adherence test replaces the tissue optimisation slide, which is specifically designed to minimise tissue detachment problems for experimental samples (Figure 1). For both workflows, four tissue adherence optimisations were successful for skin samples of different qualities, including varying storage durations and tissue sizes.

To further optimise the poly(A)‐capture method, we adapted the Visium Tissue Optimisation procedure for FFPE (manufacturer‐designed for fresh‐frozen samples) prior to library preparation (supplementary material, Figure S1). Based on the balance between capture efficiency and lateral diffusion of RNA (decreased sharpness/specificity), we determined that a permeabilisation duration of 25 min was optimal for these skin‐derived tissues. We observed that optimal conditions varied between patient samples, confirming that the tissue optimisation step was necessary prior to library preparation for the FFPE Poly(A)‐Capture protocol.

### Generating panel‐independent spatial transcriptomic data from the FFPE poly(A)‐capture protocol

**Figure 2:**
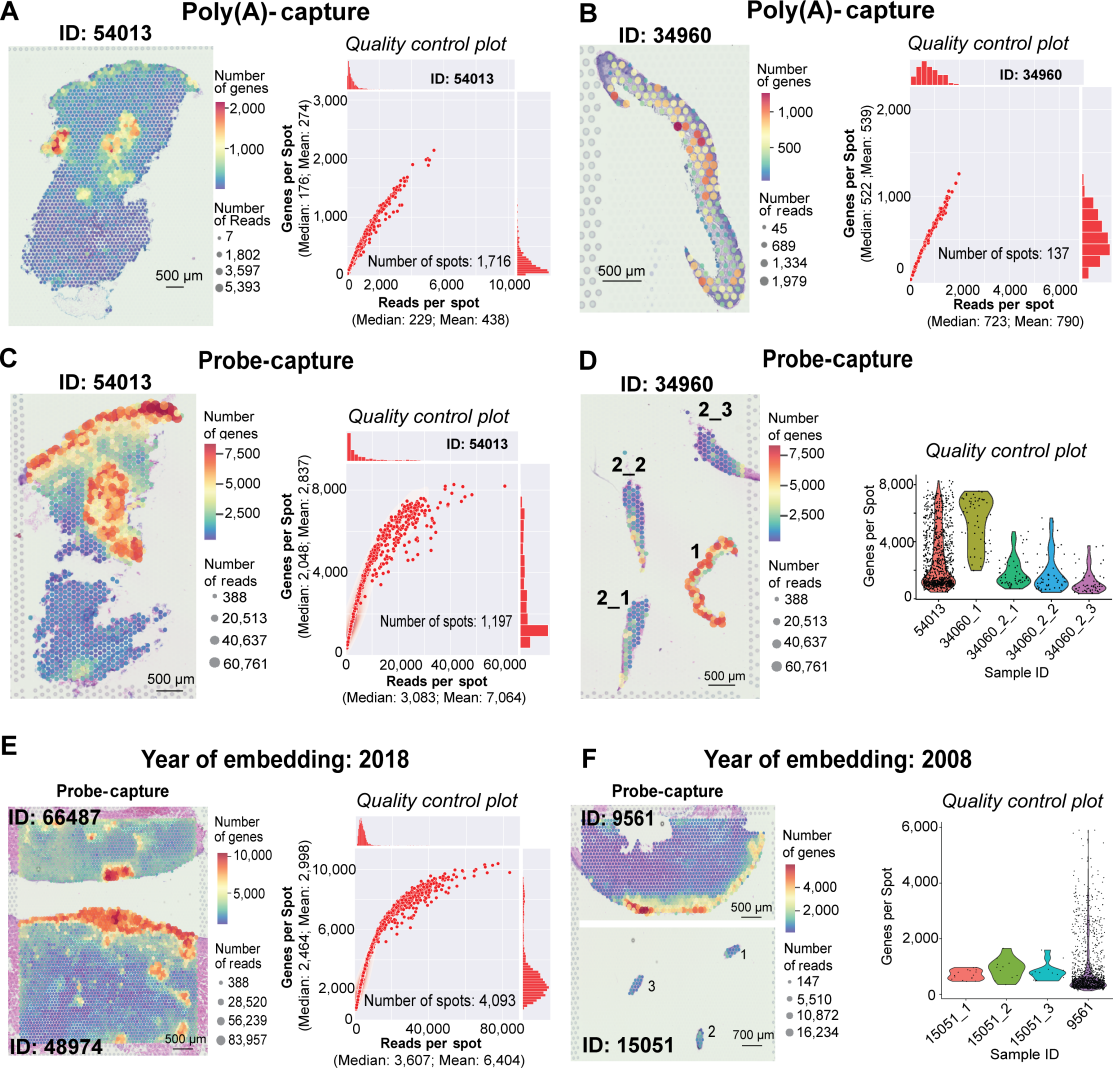
Poly(A)‐capture and probe‐capture spatial sequencing data. (A and B) The quality control (QC) for (A) the dysplastic naevus and (B) melanoma from the poly(A)‐capture protocol. (C and D) The QC for (C) the dysplastic naevus and (D) melanoma data produced by the probe‐capture method. Melanoma patient 34960 had two tissues. Tissues 34960_2 was sectioned continuously to provide triplicates on the capture area of the slide (considered as technical triplicates, labelled as 34960_2_1, 34960_2_2, and 34960_2_3). (E and F) QC for melanoma samples, which were stored for different periods of time. The melanoma patient sample 15051 had three continuous sections from the same block, considered as three replicates, which are labelled 15051_1, 15051_2, and 15051_3. The colour‐coded regions in pathological annotation images correspond to the pathologist‐drawn images in supplementary material, Figure S3.

By overlaying the ST data onto H&E images of the tissue taken early during the protocol, we visualised the number of sequencing reads and unique genes across each spatial spot and aligned these to cellular/anatomical ROIs, which suggest general statistics reflecting heterogeneity in the biological content of measured spots across the tissue (Figure 2A,B). Our optimised poly(A)‐capture workflow detected up to 2,000 genes per spot and more than 15,000 total genes per sample (Figure 2A,B), successfully generated from both large (Figure 2A; dysplastic naevus) and small (Figure 2B; melanoma) tissue sections.

### Comparing spatial transcriptomic data from probe‐capture and poly(A)‐capture protocols, assessing performance across tissue conditions and archival time

To compare the probe‐capture and poly(A)‐capture protocols, we selected the same tissue blocks for analysis (i.e. adjacent sections, patient 54,013 dysplastic naevus, and 34,960 melanoma). As expected, we observed a marked increase in the number of genes detected per spot in the probe‐capture protocol (Figure 2). For the sample replicates across each protocol, we could detect on average 2,837 genes per spot, with up to 10,000 genes per spot, higher than in the poly(A)‐capture protocol (Figure 2). We also assessed the technical reproducibility of the methods and intra‐patient variation by analysing three technical replicates (adjacent sections of the same tissue piece, 34960_2_1/2/3) and two different biopsies from the same patient (34960_1 versus 34960_2) (Figure 2C,D). The probe‐capture results were consistent across the technical replicates, demonstrated by the similar number of genes per spot (34960_2_1/2/3 and 15051_1/2/3, Figure 2D,F); inter‐replicate spot counts were much less variable than spot counts compared between different tissue sections, even those from the same patient (34960_1 versus 34960_2, Figure 2D). As expected, there was a clear disparity in the number of genes detected per spot between different biopsies of the same patient (Figure 2D), which may reflect either tumour heterogeneity or sampling variability.

Variation in storage times, storage conditions, and processing methods in retrospective studies of patient samples is a challenge as any of these factors can negatively impact RNA quality. In this project, we assessed the reproducibility of the ST methods to analyse clinical samples collected 4–14 years prior. Newer tissues (66487 and 48974, from 2018) had average DV200 scores of 70%, while older samples (9561 and 15051, from 2008) had average scores of only 31%, clearly demonstrating an impact of FFPE sample age on RNA quality. Using probe‐capture, we detected substantially more genes in the newer samples, with up to 10,000 genes per spot (Figure 2E). The older (and more degraded) samples, as anticipated, yielded a maximum of 6,000 genes per spot (Figure 2E). The data, as expected, showed that samples of lower initial RIN scores indeed yielded lower unique gene counts – a major consideration moving forward with FFPE ST. Of note, despite the reduction in gene detection sensitivity, the information from these samples remained sufficient for mapping cells consistently to histological annotation. Similar to the replicates shown in Figure 2D, we again observed consistency in quality control (QC) assessment between three adjacent replicate sections of patient 15,051 (Figure 2F), suggesting that the data from spatial profiling were reproducible even for those with low RIN scores.

### Detecting noncoding RNA from poly(A)‐capture and probe‐captured data

**Figure 3:**
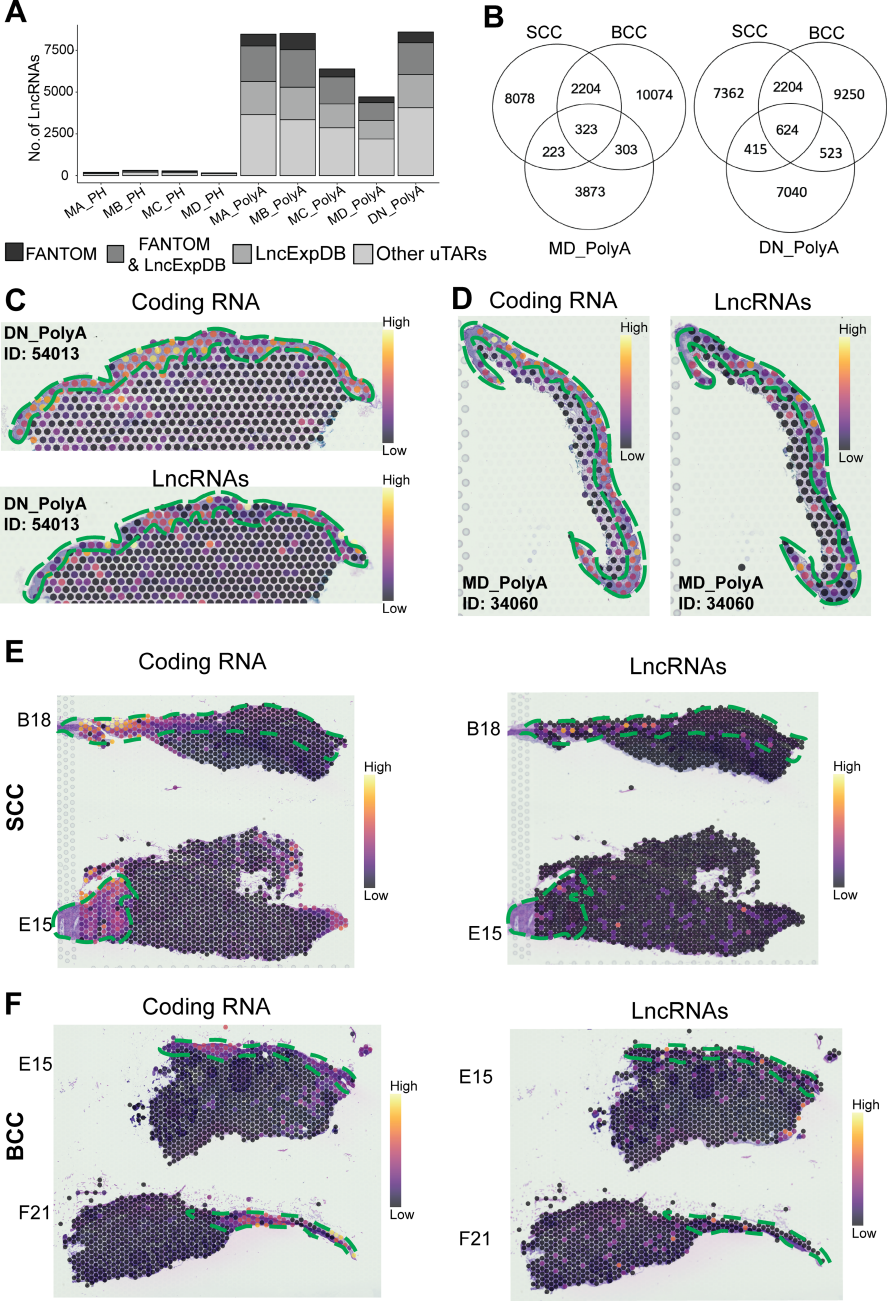
Detection of lncRNAs in melanoma and dysplastic naevus (DN) samples using polyA‐capture and probe‐capture protocols. (A) The captured lncRNAs were classified based on previously reported lncRNAs in lncExpDB and/or FANTOM protocol. Replicates are shown as MA, MB, MC, and MD, representing samples from four melanoma patients. (B) Number of uTARs that are unique or shared among melanoma/dysplastic naevus samples with fresh‐frozen BCC or SCC. (C) Top: spots on dysplastic naevus with high expression of keratinocyte markers (*KRT5, KRT1, KRT10, DSP, LOR*). Bottom: spots with significantly differentially expressed uTARs identified based on expression of keratinocyte markers. (D) Left: spots on melanoma with high expression of keratinocyte markers (*KRT5*, *KRT1*, *KRT10*, *DSP*, *LOR*). Right: spots with significantly differentially expressed uTARs identified based on expression of keratinocyte markers. (E) Left: mapping of significantly differentially expressed uTARs from (C) and (D) on fresh‐frozen SCC tissue shows mainly detection in epidermis (green dotted line). Right: spots on SCC tissues with high expression of keratinocyte markers (*KRT5*, *KRT1*, *KRT10*, *DSP*, *LOR*) showed overlap expression with identified uTARs. (F) Mapping of significantly differentially expressed uTARs from (C) and (D) on fresh‐frozen BCC tissue shows mainly detection in epidermis (green dotted line). Right: spots on BCC tissues with high expression of keratinocyte markers (*KRT5*, *KRT1*, *KRT10*, *DSP*, *LOR*) show overlap expression with identified uTARs. BCC, basal cell carcinoma; lncRNAs, long non‐coding RNA; MA, melanoma A; MB, melanoma B; MC, melanoma C; MD, melanoma D; PH, probe‐capture; polyA, poly(A)‐capture; SCC, squamous cell carcinoma; uTAR, unannotated transcribed adenylate region.

While most analyses for spatial transcriptomics data have focused on protein coding genes, there is huge potential to detect lncRNAs in tissue. Here we report one of the first results on the spatial expression of lncRNA and the association of lncRNA spatial expression patterns with morphological features. Analysing multiple replicates, we found that the poly(A)‐capture protocol detected a large number of lncRNAs (>9,000 lncRNAs per sample) (Figure 3A). Importantly, more than 50% of the detected lncRNAs were also present in two well‐curated lncRNA databases, LncExpDB and FANTOM, suggesting that the lncRNAs in the poly(A)‐capture spatial datasets were mostly true lncRNAs. The presence of remaining uncharacterised sequences presents an opportunity for further discovery and reflects the evolving nature of lncRNA research. This finding highlights the potential to expand our knowledge of lncRNA diversity and the value of using comprehensive datasets to advance our understanding of these molecules. Thus, the poly(A)‐capture protocol detected fewer coding genes in total but identified a significant number of lncRNAs that were not detected through probe‐capture. Future work can explore the design of probes for lncRNAs; however, this approach has limitations, including challenges with probe sensitivity, non‐specific binding, high costs, and the incomplete characterisation of many lncRNAs, especially those specific to certain tissues or disease conditions. Overall, our results suggest a complementarity between the two protocols and that the Poly(A)‐Capture protocol can fill an important gap inherent in the probe‐capture protocol.

In further investigations of lncRNAs in FFPE tissues, we compared them with those found in fresh‐frozen samples. We detected more than 300 lncRNAs in melanoma and 600 lncRNAs in dysplastic naevi that were also detected when calling uTARs from ST data of fresh‐frozen skin cancer samples [basal cell carcinoma (BCC) and squamous cell carcinoma (SCC)] (Figure 3B). We then compared the expression of uTARs in keratinocytes and identified eight significantly differentially expressed uTARs in the keratinocytes of dysplastic naevus and 10 uTARs in the keratinocytes of melanoma (Figure 3C,D). The expression patterns of these uTARs appeared to correlate with the distribution of canonical keratinocyte markers, suggesting potential cell‐type‐specific functions. It is worth noting that further investigation is required to explore the functionality of these uTARs through the detection and comparison of their expression across multiple samples. Comparing with uTARs detected in fresh‐frozen SCC and BCC tissues (Figure 3E,F), of the 18 uTARs examined, six were detected in SCC tissues and seven in BCC tissues. Notably, seven of the 18 uTARs were novel/unreported, while the remaining have been reported as lncRNAs in other databases such as LncExpDB (LncBook), LNCpedia, and FANTOM (supplementary material, Table S3), indicating the validity of these uTARs.

### Characterising heterogeneity within FFPE skin cancer tissues

We found that the spatial transcriptomics data of the FFPE samples (storage 4–14 years) could be used to accurately map cell types to the tissue at a detailed resolution, allowing for unprecedented quantification of heterogeneity. Here we assessed two skin disease stages, dysplastic naevus and melanoma. Three technical replicates as consecutive sections from the same block were included to assess technical variation.

**Figure 4:**
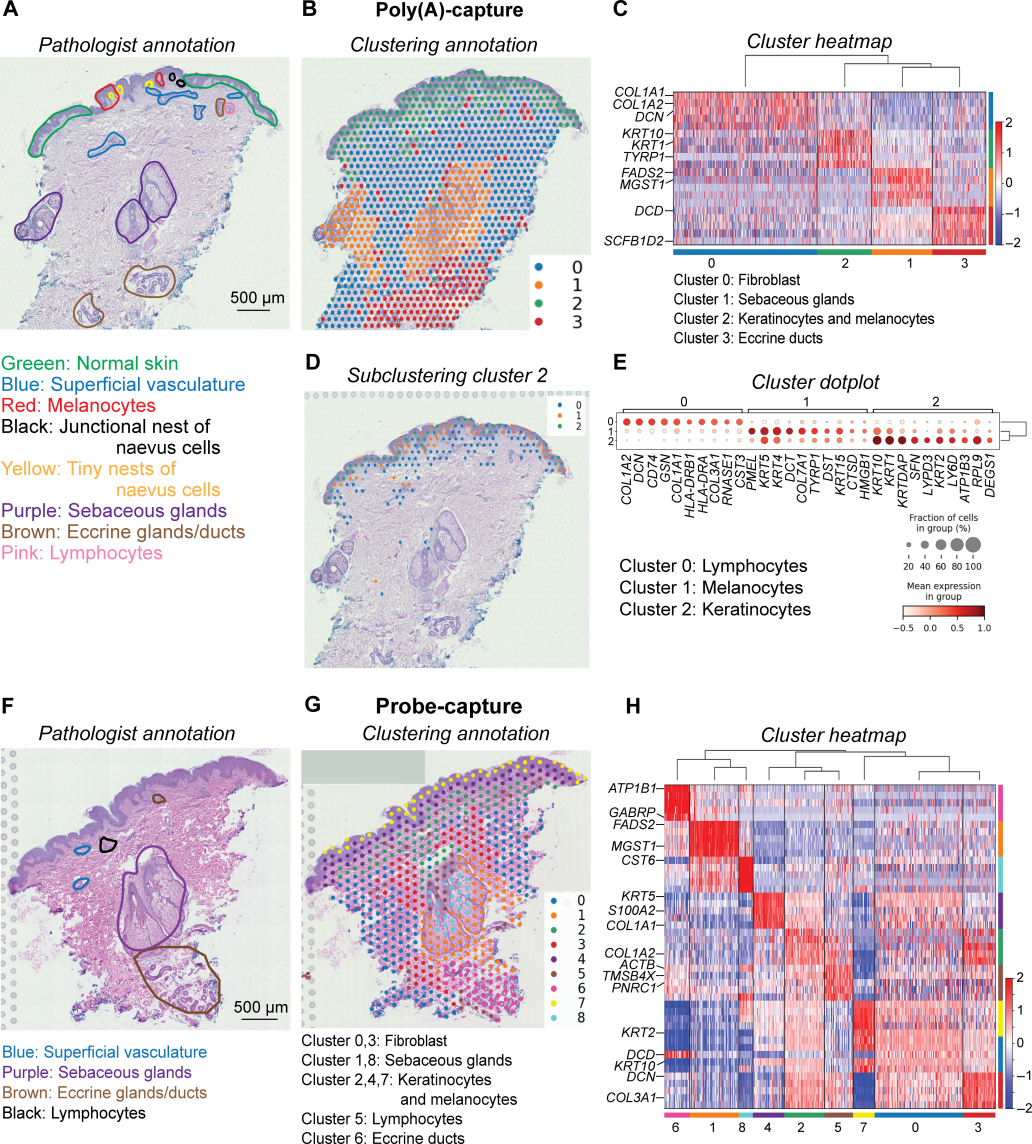
Data‐driven, unsupervised mapping of heterogeneous tissue regions across dysplastic naevus tissue sections. (A) Annotation by pathologist of dysplastic naevus from poly(A)‐capture. Pathologist annotation is shown as coloured circles. (B) Spatial clustering and (C) heatmap of dysplastic naevus from poly(A)‐capture revealed molecularly defined clusters that are heterogeneous and consistent with pathological annotation. (D) Spatial subclustering and (E) heatmap of cluster 2 defined in first‐round clustering of dysplastic naevus (as shown in B and C). (F) Annotation of dysplastic naevus from probe‐capture by pathologist. Pathologist annotation is shown as coloured circles. (G and H) The clustering of dysplastic naevus tissue from (G) probe‐capture shows more heterogeneity details. (H) Heatmap shows top gene markers for each cluster. The colour‐coded regions in the pathological annotation images correspond to the pathologist‐drawn images in supplementary material, Figure S3.

Heterogeneity was assessed through manual pathologist annotation and by unsupervised spatial clustering at spot level (one spot contains one to nine cells), revealing a high level of heterogeneity within a dysplastic naevus skin tissue section (Figure 4A–H and supplementary material, Figure S3A,B). We identified four clusters in poly(A)‐capture data and nine clusters in probe‐capture data that overall match the manually annotated tissue types. In poly(A)‐capture data, we defined genes associated with fibroblasts producing collagen in the dermis (with markers *COL1A1, COL1A2, DCN*), along with adnexal structures such as sebaceous glands (*FADS2, MGST1*), eccrine ducts (*DCD, SCFB1D2*). Importantly, genes marking common epidermal cells were identified and mapped to areas enriched with keratinocytes and melanocytes (*KRT10, KRT1, TYRP1*) (Figure 4B,C, and supplementary material, Table S4). In probe‐capture data, we were able to detect more lymphocytes (cluster 5 – *ACTB, TMSB4X, PNRC1*) (Figure 4G,H, and supplementary material, Table S5) within the sebaceous gland clusters and eccrine duct clusters. Of note, in the poly(A)‐capture data, by subclustering cluster 2 (keratinocytes and melanocytes), we were able to find lymphocytes (*CD74, HLA‐DRB1, HLA‐DRA*) and melanocytes (*PMEL, DCT, TYRP1*) (Figure 4D,E, and supplementary material, Table S6). Based on the expression profiles of over 15,000 genes across the whole tissue section (up to 5,000 spots per tissue), we identified 19 molecularly distinct cell types or functional regions for the dysplastic naevus sample (supplementary material, Figure S4A). These cell types and regions showed spatially specific expression of gene markers, for example the pigment‐cell (melanocyte)‐specific premelanosome gene (*PMEL*) encoding melanocyte‐specific type I transmembrane protein. Visual inspection of *PMEL* gene expression also suggested that *PMEL* was expressed in the naevus region (and supplementary material, Figure S4B). Less known marker genes specific to a cell type or functional region, such as *PRDX2*, could also be detected (and supplementary material, Figures S3 and S4C).

**Figure 5:**
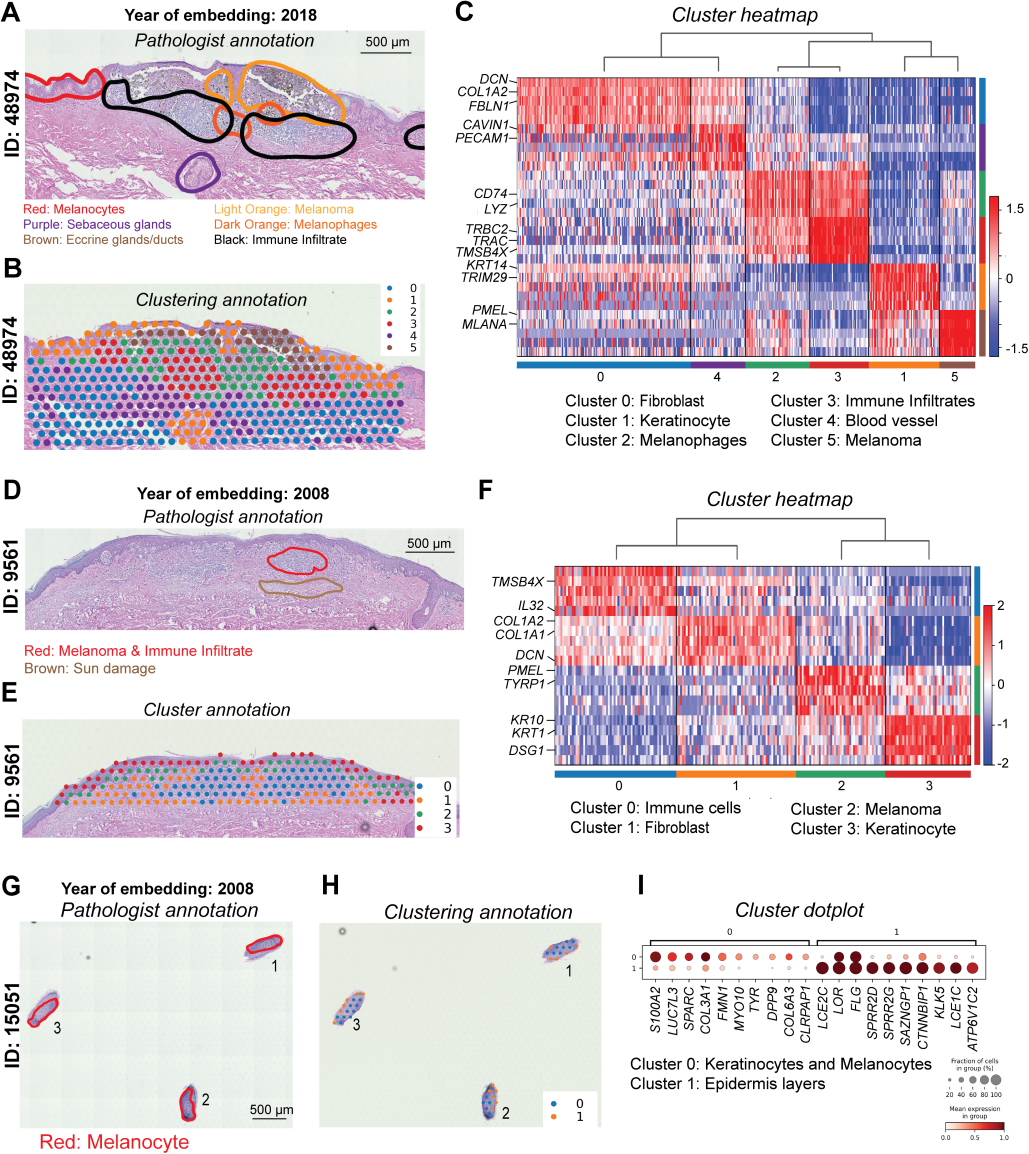
Visium probe‐capture for melanoma FFPE samples stored at different periods of time. (A, D, and G) Pathologist annotation as coloured circles. (B, E, and H) Corresponding clustering results from tissues in A, D, and G, respectively. (C, F, and I) Heatmaps of gene marker expression for each cluster in B, E, and H, respectively.

For the melanoma samples (Figure 5), the data for patient ID 48974, which were collected in 2018, contain six main clusters. Gene markers for these clusters, as shown in the heatmap, suggest cell‐type annotation consistent with pathologist‐determined tissue regions (Figure 5A–C, and supplementary material, Figure S5A and Table S7). We defined clusters corresponding to melanoma (*PMEL, MLANA*), immune infiltrates (*TRBC2, TRAC, TMSB4X*), melanophages (*CD74, LYZ*), keratinocytes (*KRT14, TRIM29*), blood vessels (*CAVIN1, PECAM*), and fibroblast‐enriched areas in the dermis (*DCN, COL1A2, FBLN1*). Depending on tissue sizes and complexity, the number of clusters changed. A smaller tissue from patient ID 9561, collected in 2008, had four clusters, including melanoma (*PMEL, TYRP1*), immune cells (*TMSB4X, IL32*), keratinocytes (*KRT10, KRT1, DSG1*), and fibroblast areas (*COL1A2, COL1A1, DCN*) (Figure 5D–F, and supplementary material, Figure S5B and Table S8). For the smallest tissue from patient ID 15051, with three biological replicates, there were two specific clusters consistently defined across the replicates. These two clusters were keratinocytes and melanocytes (cluster 0 – *S100A2, SPARC, TYR, MYO10*) versus epidermis (cluster 1 – *LCE2C, FLG*), consistent with the pathological annotation (Figure 5G–I and supplementary material, Figure S5C and Table S9).

Together, our spatial data showed strong evidence that the spatial gene expression was able to capture tissue heterogeneity at a high resolution (higher than pathological annotation), across the whole tissue section and in an automated and unbiased way.

**Figure 6:**
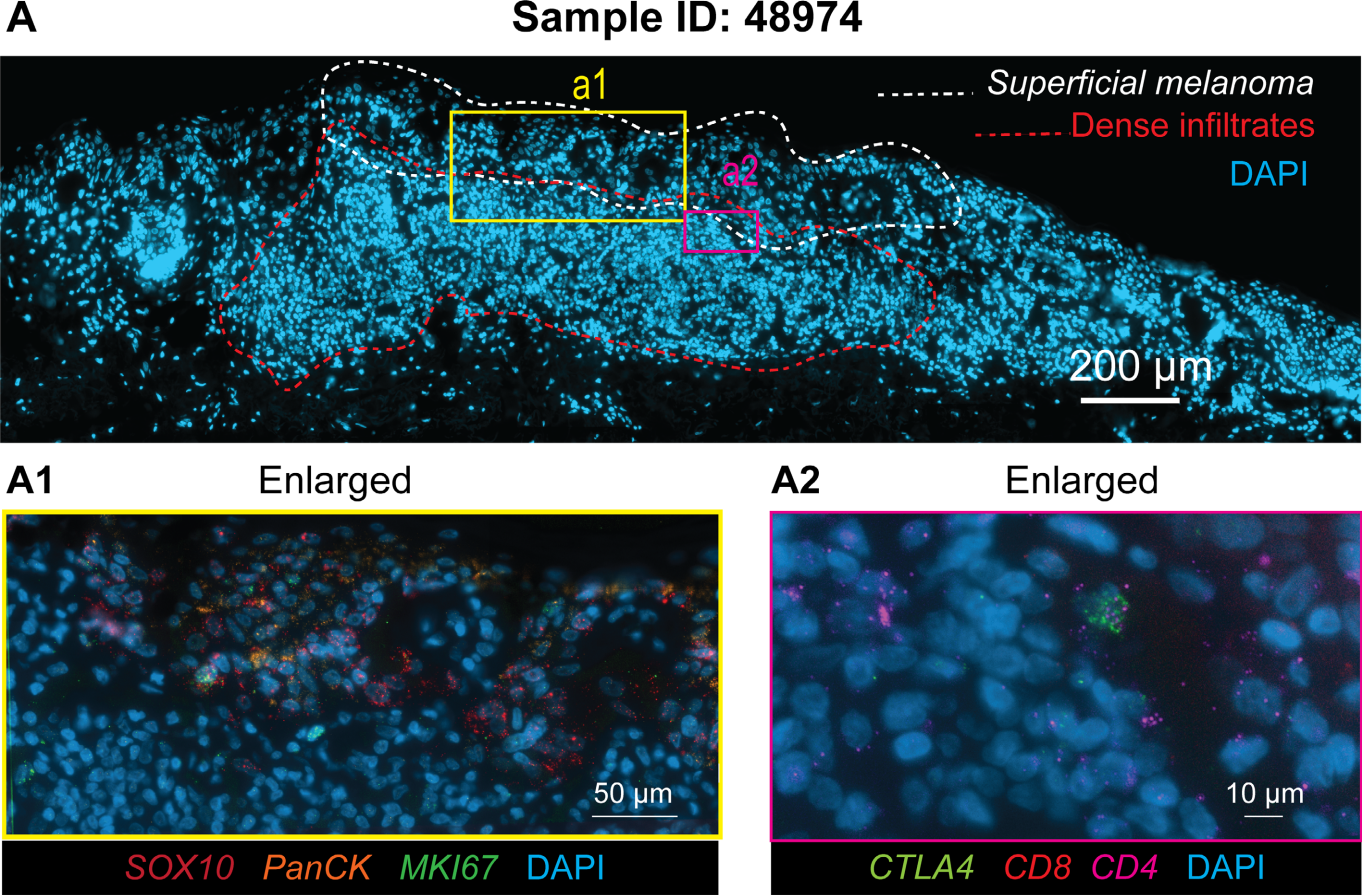
Targeted imaging of RNA expression at a single cell level using RNAScope assay. (A) Overview of section with nuclei stained with DAPI and pathological annotation circled by white and red lines. Zoomed‐in shot of a superficial melanoma region, showing two windows, A1 and A2. (A1) Cancer markers *SOX10, PanCK*, and *MKI67*. These genes are expressed in the melanoma metastasis region near the epithelial layers. Each punctate dot represents a single copy of a mRNA molecule. (A2) Expression of *CD4* T‐cell marker (*CD4, CTL4A*) and CD8 T cell is observed in immune cell infiltration area. The unsharp mask filter was used to enhance the apparent sharpness of an image.

Moreover, we also assessed imaging‐based methods to measure RNA expression at single‐cell resolution in FFPE tissue sections. We performed an RNAScope assay, which has high sensitivity but is only able to detect a small set of genes (up to 12 molecules) (Figure 6, and supplementary material, Figure S6). We selected six genes as known markers of cancer cells and immune cells in skin cancer to assess tissue heterogeneity and to compare with ST data. As with the ST experiment, we also provided the pathological annotation based on nucleus shapes and distribution from the same slide, defining immune infiltration and superficial melanoma regions (and supplementary material, Figure S6A). Each punctate dot signal on a cell represents a single molecule in the assay. As a result, the assay established the abundant expression of the melanoma marker gene *SOX10* in the superficial melanoma region along with its co‐expression with the proliferation‐associated *MKI67* (and supplementary material, Figure S6A1). Also, the distinct co‐expression of *CD4* and *CTLA4* was seen in the immune cell infiltrate area with a low expression of *CD8* (and supplementary material, Figure S6A2). Compared to pathological annotation, it appears that both ST and RNAScope can more precisely define the cancer and immune regions. ST yielded much more information on molecular expression profiles that mark individual cell types and activities. While RNAScope provides single‐cell resolutions and high detection sensitivity, the ST generated data for thousands of times more genes that enabled identification of individual cell types, cell states, and their activities across the tissue section.

Further, we also assessed heterogeneity at the protein level, with the application of the highly multiplexed spatial proteomics technique for a similar set of FFPE tissue melanoma samples used for ST data. We observed that CODEX could detect more protein targets than traditional IF, but data for cell‐type mapping could be noisy or missing, for example in the case of KRT8, PMEL, HLA, Ki67, and CD278 (and supplementary material, Figures S7 and S8). As with conventional IF staining, large autofluorescence regions were also observed in CODEX (see supplementary material, Figures S7,S8). For comparison between CODEX and ST data regarding cell‐type markers, cell‐type deconvolution methods were first applied to the ST data (see supplementary material, Figure S7A,B). The spatial deconvolution of single‐cell RNA sequencing data was consistent with pathological annotation (and supplementary material, Figure S7B). We also observed that ST mapped cell types to more discrete/defined tissue regions, while the protein signal in CODEX was more diffuse (e.g. PMEL for melanocytes in supplementary material, Figure S8 and CD4 markers for immune cells in supplementary material, Figure S7).

## Discussion

Archived FFPE tissue samples, a worldwide standard in pathology departments, provide an invaluable resource for molecular and translational research, but the enormous number of tissue collections in biobanks is underutilised [38, 39, 40, 41]. Spatial methods for FFPE tissues, therefore, are powerful and necessary tools to enable breakthroughs in understanding pathological processes in cancer tissue, especially regarding tumour heterogeneity. Despite the vast potential for pathological applications, ST has not been popular for these samples due to nucleic acid crosslinking, molecular degradation, and tissue‐slide detachment [8, 41]. In this study, we established two alternate ST methods to overcome these technical challenges with FFPE tissues. To assess the breadth and generalisability of these tools, we evaluated tissues of variable sizes, archival times, cancer progression level, and RNA quality across biological and technical replicates. We further assessed other imaging‐based spatial methods for FFPE skin cancer tissues, including RNAScope and CODEX.

In clinical practice, manual observation of FFPE melanoma tissues by pathologists is often limited to a small number of cell types and anatomical regions, leading to incomplete assessment of tumour heterogeneity. This in turn limits accurate diagnosis, which is the basis for effective treatment plans [42, 43, 44, 45, 46, 47]. Current common spatial techniques can, on average, detect less than 100 proteins and fewer than 500 gene markers. Comparatively, Visium is a ST technology capable of measuring the spatial whole transcriptome and near single‐cell resolution while at the same time generating histological‐grade tissue images. We optimised the poly(A)‐capture protocol as this method can capture RNAs that are not in a predefined probe set, thereby providing missing information such as lncRNA expression or detection of RNA from species without predesigned probes. The gene detection capacity of the two FFPE protocols reported here can be thousands of times higher than classic pathology techniques such as H&E and IHC. The probe‐capture protocol detected more genes with increased sensitivity but missed genes not in the panel, especially lncRNAs. Our poly(A)‐capture protocol, in contrast, enables detection of thousands of lncRNAs. This additional information is important as lncRNAs play an important role in melanoma development, including proliferation, invasion, and apoptosis [48]. The detailed roles and impacts of these lncRNAs were described in a recent publication, highlighting the advantage of this poly(A)‐capture protocol [49]. Moreover, the application of our poly(A)‐capture protocol also allows for patient‐derived xenograft, non‐mouse tissue, or non‐human tissue, where no probe panel is available. This is an example of the usefulness of performing ST on FFPE samples, without having to depend on a panel. We expect that many more applications can be derived from this protocol.

Our protocols worked with challenging FFPE skin tissues older than 14 years old with high degradation (DV200 < 30%) (Figures 1, 5, 6). We have tested numerous sectioning and storage conditions and optimised section thickness to improve section adhesion [8] preserving both tissue adherence and RNA quality. Moreover, since cost is a major barrier to applying ST in both translational research and clinical practice, we also validated the option to multiplex tissue samples into Visium capture arrays for space maximisation. In this way, we were able to analyse up to nine tissue sections per slide, rather than an average of four samples per slide as in a standard protocol.

From our thorough assessment of these protocols, we suggest that for discovery beyond the coding genes included in the human or mouse Visium panels, an unbiased FFPE poly(A)‐capture approach can be useful, as it is capable of detecting a broader range of genes, including lncRNA and RNAs from different species such as in the non‐human or non‐mouse studies [50] or in patient‐derived xenograft models. By comparing multiple replicates, we found that both protocols had high reproducibility, with much less technical variation compared to biological differences. This suggests that, going forward, biological replicates will be more relevant than technical replicates and that a larger number of biological replicates will be necessary to better characterise cancer heterogeneity. We also demonstrated a practical multiplexing strategy to reduce costs, which makes it possible to increase sample sizes. For low‐throughput confirmation of results, we suggest using RNAScope with high sensitivity and resolution. Our comprehensive results reported here provide empirical support to the community for processing old/degraded FFPE tissues to generate thousands of times richer information in terms of spatial data, opening new horizons to explore cancer tissue biology.

## Author contributions statement

QN, MSS, KK and TV conceived the study. TV, KJ, SY, PYL, JC and NM performed the experiments with the help of SW. QN, TV, PP, IG and XJ performed data analysis. Y‐CK, CZ, KK and MSS provided samples and ethics management. PHS annotated H&E‐stained tissue sections. QN, TV, KJ, PP, SY, MSS, NM and SXT wrote the manuscript. All authors read and approved the manuscript.

## Supporting information


**Figure S1.** Tissue optimisation experiment performed prior to poly(A)‐capture workflow
**Figure S2.** RNA quality assessment of dysplastic naevi and melanoma samples
**Figure S3.** Pathological annotation of dysplastic naevus section used in poly(A)‐capture protocol and probe‐capture protocol
**Figure S4.** Spatial heterogeneity at gene level
**Figure S5.** Pathological annotation of melanoma tissue sections used in this article
**Figure S6.** Comparison of pathologist annotation, Visium clustering, and RNAScope assay
**Figure S7.** Comparative analysis of cell‐type detection using Visium and protein profiling with CODEX
**Figure S8.** Comparison of gene detection using Visium and protein detection using CODEX

**Table S1.** Information for sample tissues
**Table S2.** Pre‐oligonucleotide‐conjugated antibodies and complementary reporters
**Table S3.** Significantly differentially expressed uTARs in keratinocytes of dysplastic naevus and 10 uTARs in keratinocytes of melanoma
**Table S4.** Top 100 genes per cluster in dysplastic naevus from poly(A)‐capture data – related to Figure 5B
**Table S5.** Top 100 genes per cluster in dysplastic naevus from probe‐capture data – related to Figure 5G
**Table S6.** Top 100 genes per subcluster in dysplastic naevus from poly(A)‐capture data – related to Figure 5D
**Table S7.** Top 100 genes per cluster in melanoma ID 48974 – related to Figure 6B
**Table S8.** Top 100 genes per cluster in melanoma ID9561 – related to Figure 6E
**Table S9.** Top 100 genes per cluster in melanoma ID 15051 – related to Figure 6H

## Data Availability

Datasets supporting this manuscript are available at Zenodo, DOI: https://doi.org/10.5281/zenodo.8053920 and code supporting this manuscript used our stLearn spatial transcriptomics analysis software available at https://stlearn.readthedocs.io/en/latest/.
