# Supplementary material for: Assessing spatial sequencing and imaging approaches to capture the molecular and pathological heterogeneity of archived cancer tissues
Source: J Pathol. 2025 Jan 23;265(3):274–88. doi: 10.1002/path.6383 (PMC11794982; doi:10.1002/path.6383)
Supplement: Supplementary file 1 — Figure S1. Tissue optimisation experiment performed prior to poly(A)‐capture workflow Figure S2. RNA quality assessment of dysplastic naevi and melanoma samples Figure S3. Pathological annotation of dysplastic naevus section used in poly(A)‐capture protocol and probe‐capture protocol Figure S4. Spatial heterogeneity at gene level Figure S5. Pathological annotation of melanoma tissue sections used in this article Figure S6. Comparison of pathologist annotation, Visium clustering, and RNAScope assay Figure S7. Comparative analysis of cell‐type detection using Visium and protein profiling with CODEX Figure S8. Comparison of gene detection using Visium and protein detection using CODEX [file PATH-265-274-s001.docx]

**Assessing spatial sequencing and imaging approaches to capture the molecular and pathological heterogeneity of archived cancer tissues**

T Vo *et al. J Pathol* <https://doi.org/10.1002/path.6383>

**Supplementary Figures S1–S8**


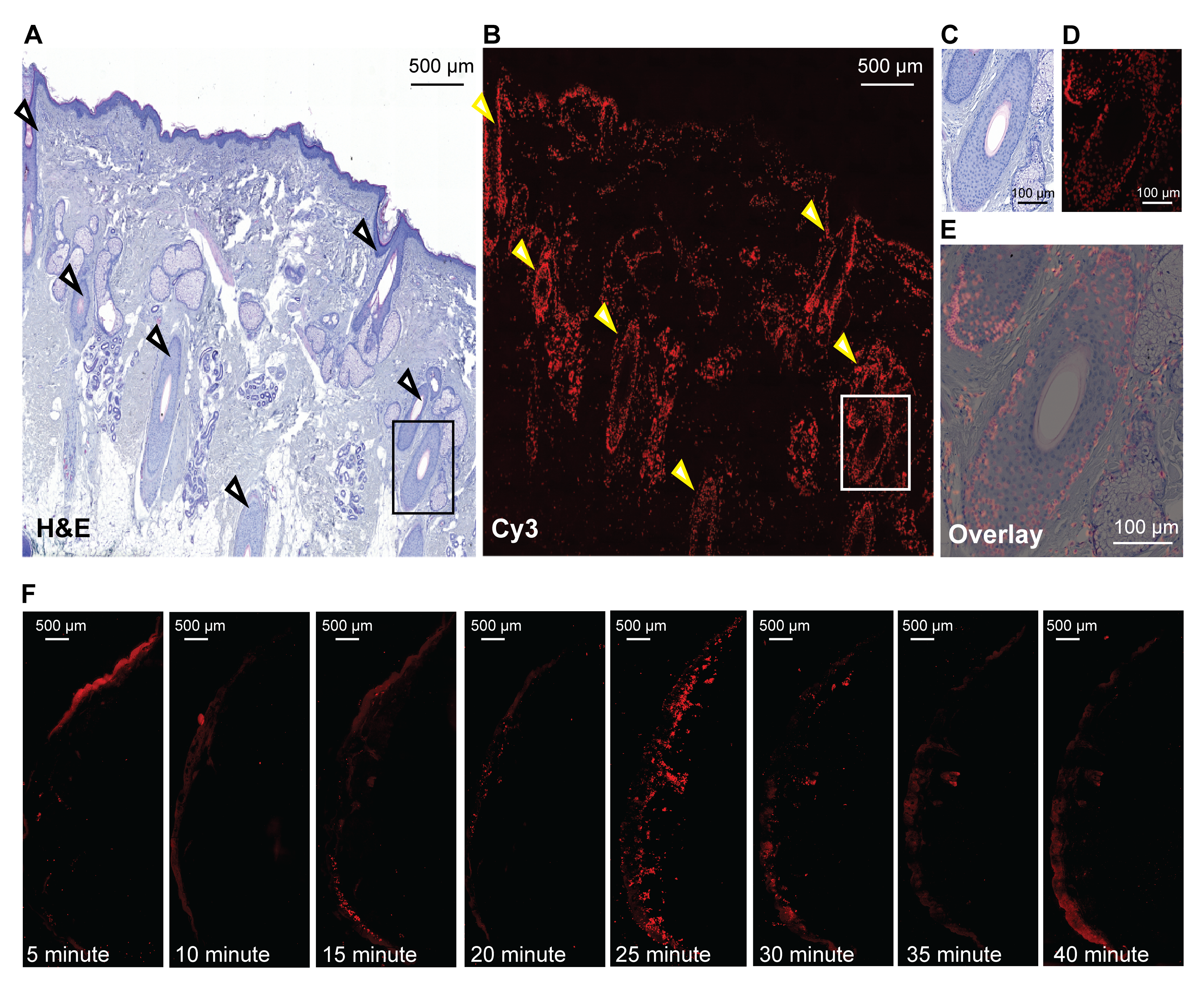


**Figure S1. Tissue optimisation experiment performed prior to poly(A)-capture workflow.** (A) Brightfield (H&E-stained) image of the tissue section. (B) Fluorescent (Cy3-tagged, poly(dT)-bound cDNA) image of the tissue section. (C) Box denotes an enlarged region on the brightfield image. (D) Box denotes an enlarged region on the fluorescent image. (E). Overlays can be used as a measure of quality control by assessing that Cy3 signal is consistent to H&E morphology, with cDNA concentrated to the densely nucleated follicular tissue. (F) Cy3 images as a time series of tissue section permeabilisations, beginning with 5 min and proceeding to 40 min (incubation with 0.1% pepsin). The 25 min permeabilisation was chosen as optimal from the series, with highly concentrated poly(dT)-bound cDNA evidenced as the most intense and tissue-specific Cy3 signal.

***
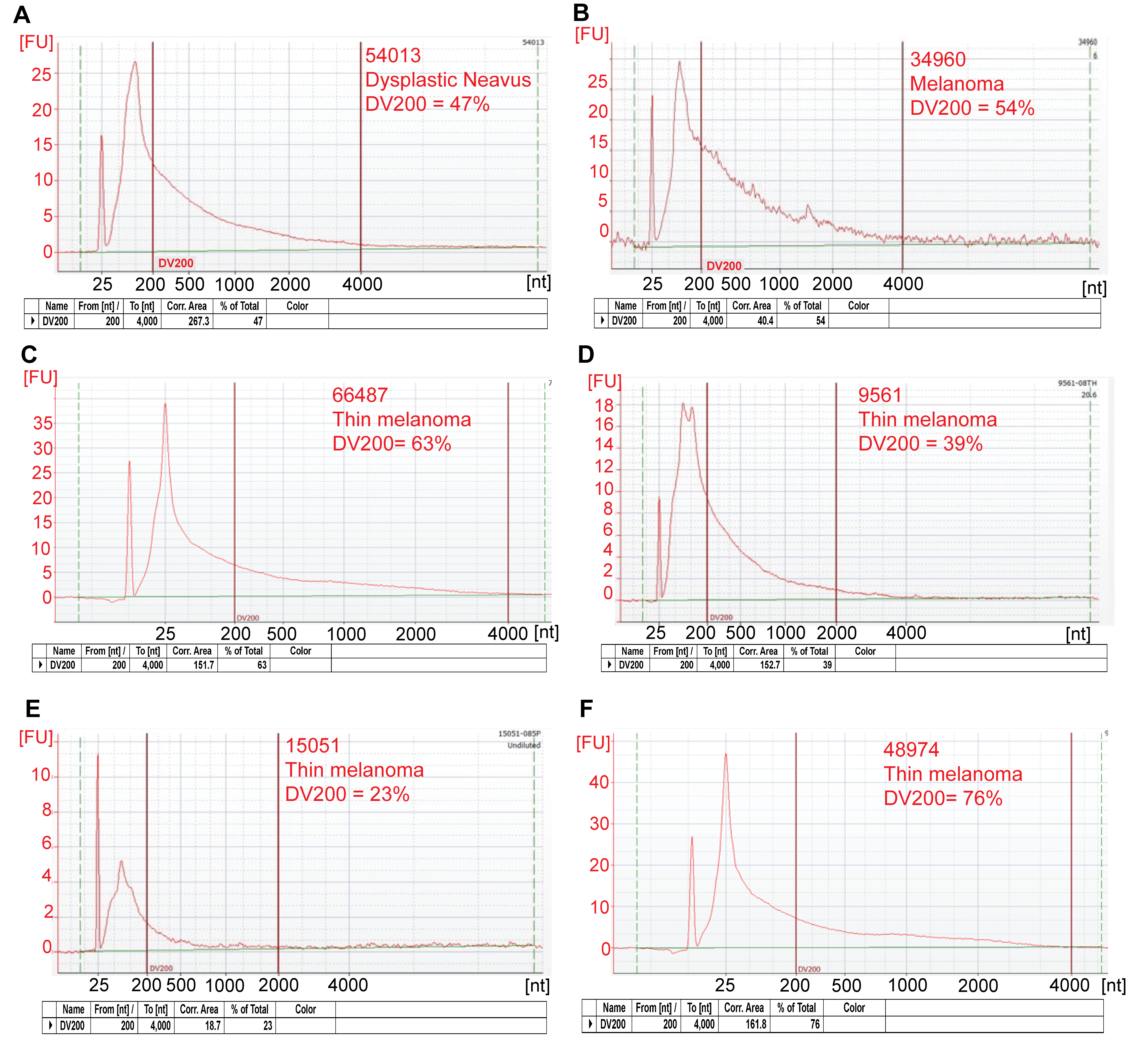
***

**Figure S2. RNA quality assessment of dysplastic naevi and melanoma samples.** RNA quality (DV200) for dysplastic naevi and melanoma samples, where DV200 represents the percentage of RNA fragments above 200 nucleotides The electropherograms show the RNA fragment distribution in each sample, with the red lines representing RNA fragment size (x-axis) and the quantity or intensity (y-axis). The vertical red lines indicate the 200-nucleotide threshold used to calculate the DV200 values. (A) Sample 54013, Dysplastic Nevus, DV200 = 47%. (B) Sample 34960, Melanoma, DV200 = 54%. (C) Sample 66487, Thin Melanoma, DV200 = 63%. (D) Sample 9561, Thin Melanoma, DV200 = 39%. (E) Sample 15051, Thin Melanoma, DV200 = 23%. (F) Sample 48974, Thin Melanoma, DV200 = 76%. Higher DV200 values indicate a higher proportion of longer RNA fragments, suggesting better RNA integrity.


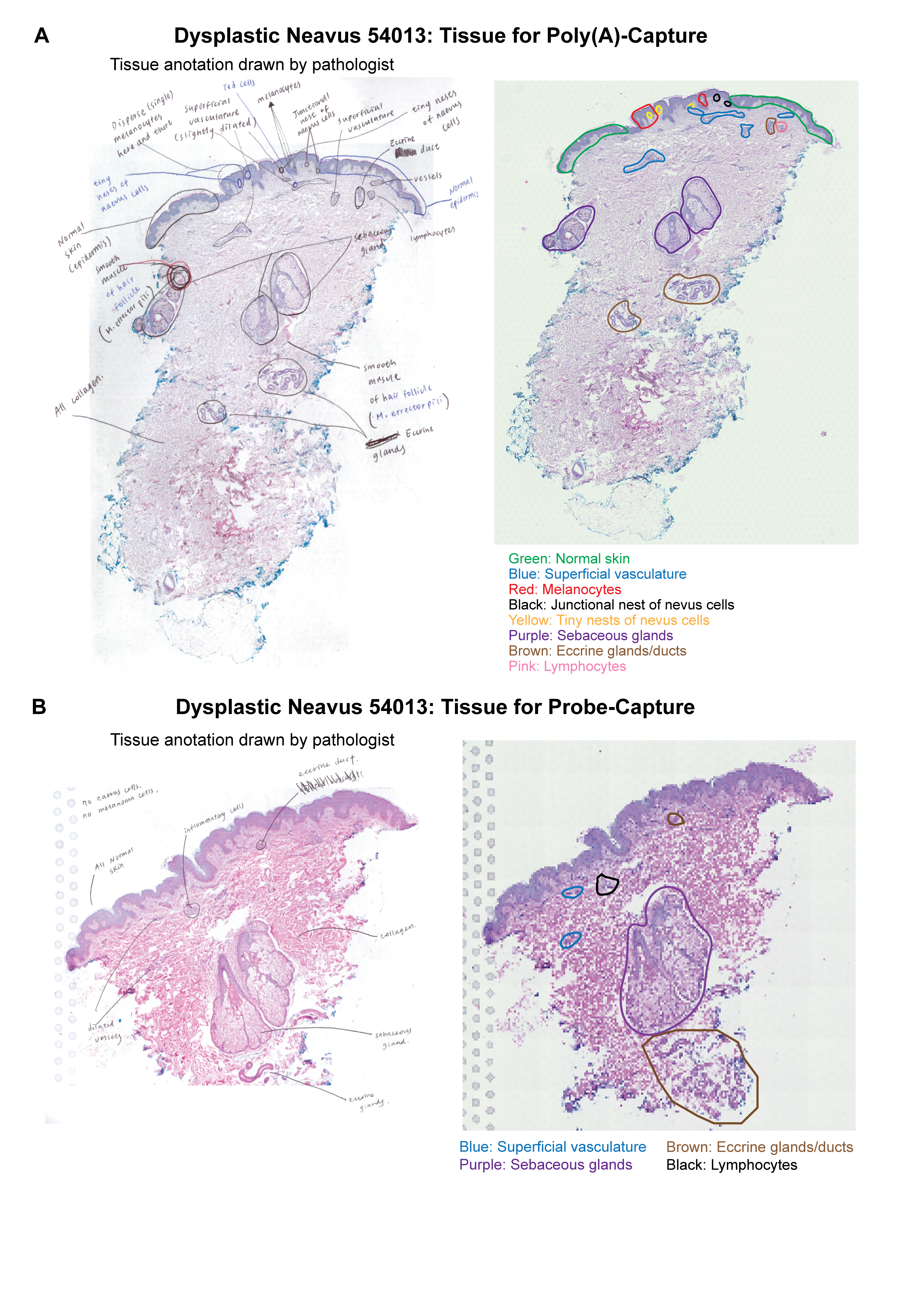


**Figure S3. The pathological annotation of the dysplastic naevus section used in poly(A)-capture protocol and probe-capture protocol.** (A) Dysplastic naevus section used for poly(A)-capture protocol. Left is the original annotation and right is the transfer of the selected regions with colour coding. (B) Dysplastic naevus section from the same block, but was cut deeper, used for probe-capture protocol. The annotation from left is transferred to the right with colour codes. The colour-coded pathological annotation images are used in Figure 4, and supplementary material, Figures S4,S8 to compare with the gene expression clustering annotation and deconvolution annotation.


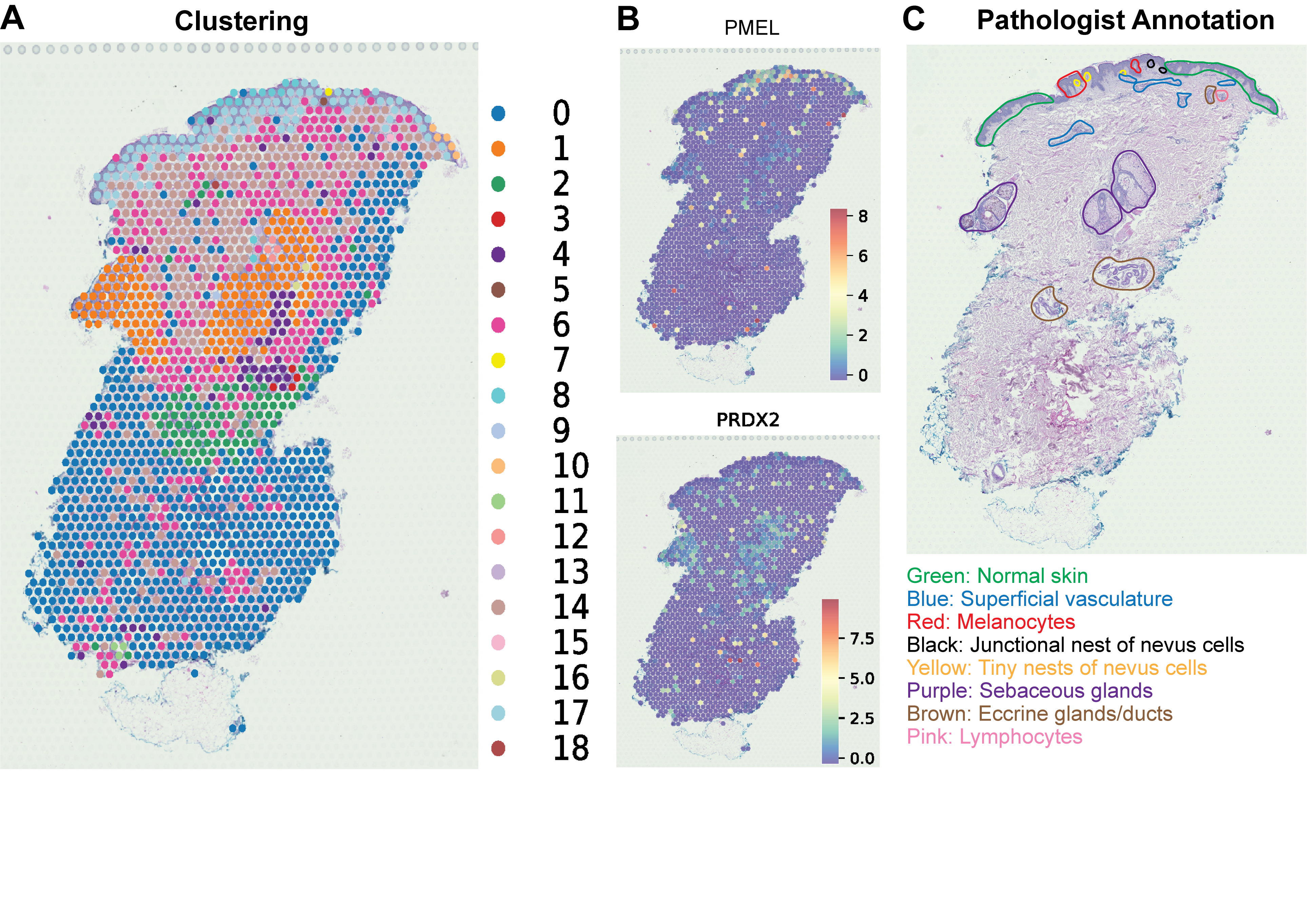


**Figure S4. Spatial heterogeneity at the gene level**. The clustering results are shown on the left, histopathological on the right. The heatmap gradient colours in the middle show the expression level of two melanoma markers across the tissue section. The colour-coded regions in pathological annotation images correspond to the pathologist-drawn images in supplementary material, Figure S3.


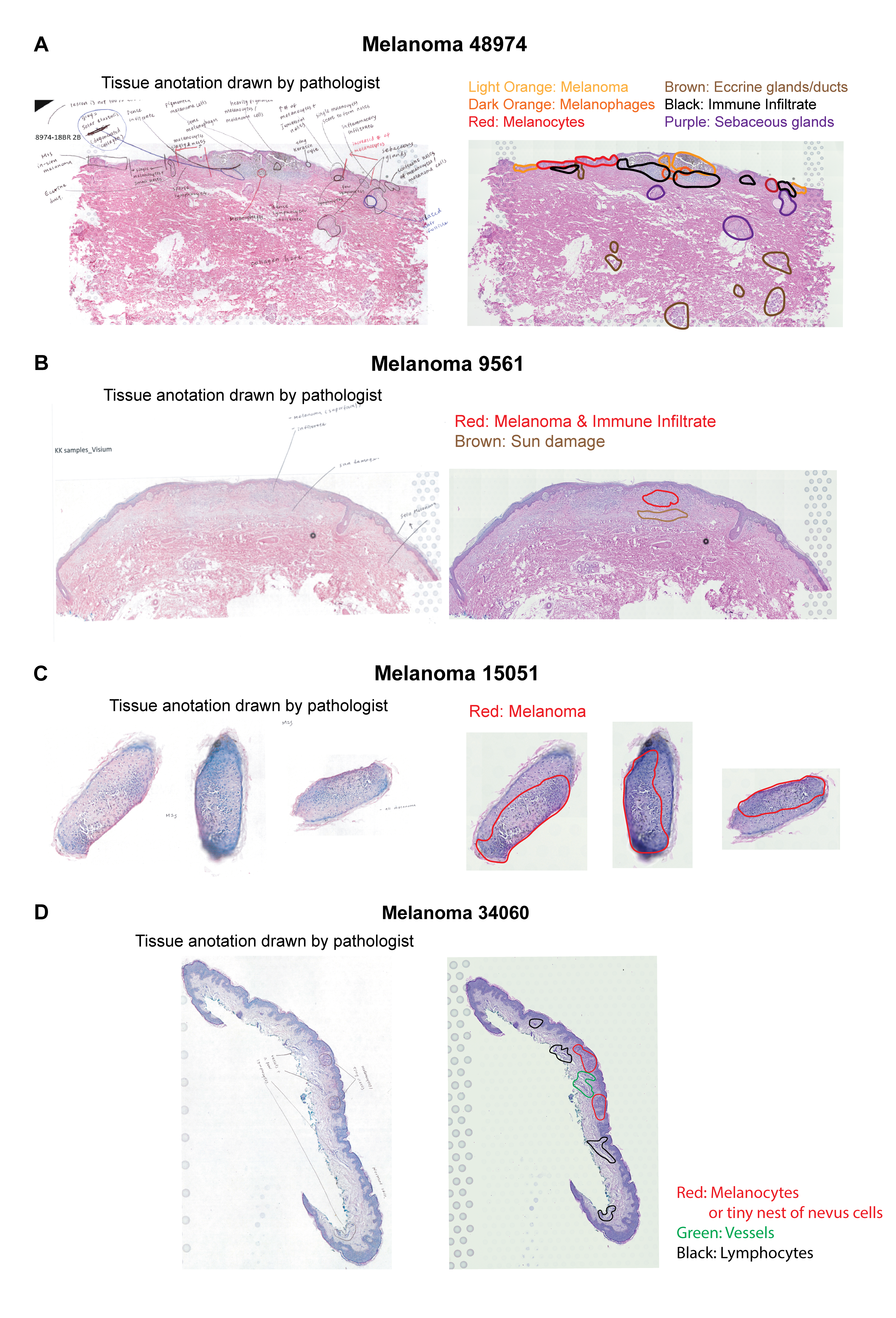


**Figure S5. The pathological annotation of the melanoma tissue sections used in this article.** (A) Annotation for patient 48974. The six regions are colour-coded and transferred from left to right. (B) Annotation for patient 9561. The annotated melanoma and sun-damaged regions are transferred from left to right images. (C) Annotation for patient 15051. Three sections are three technical replicates. The colour-coded pathological annotation images are used in Figure 5 to compare with the gene expression clustering annotation.


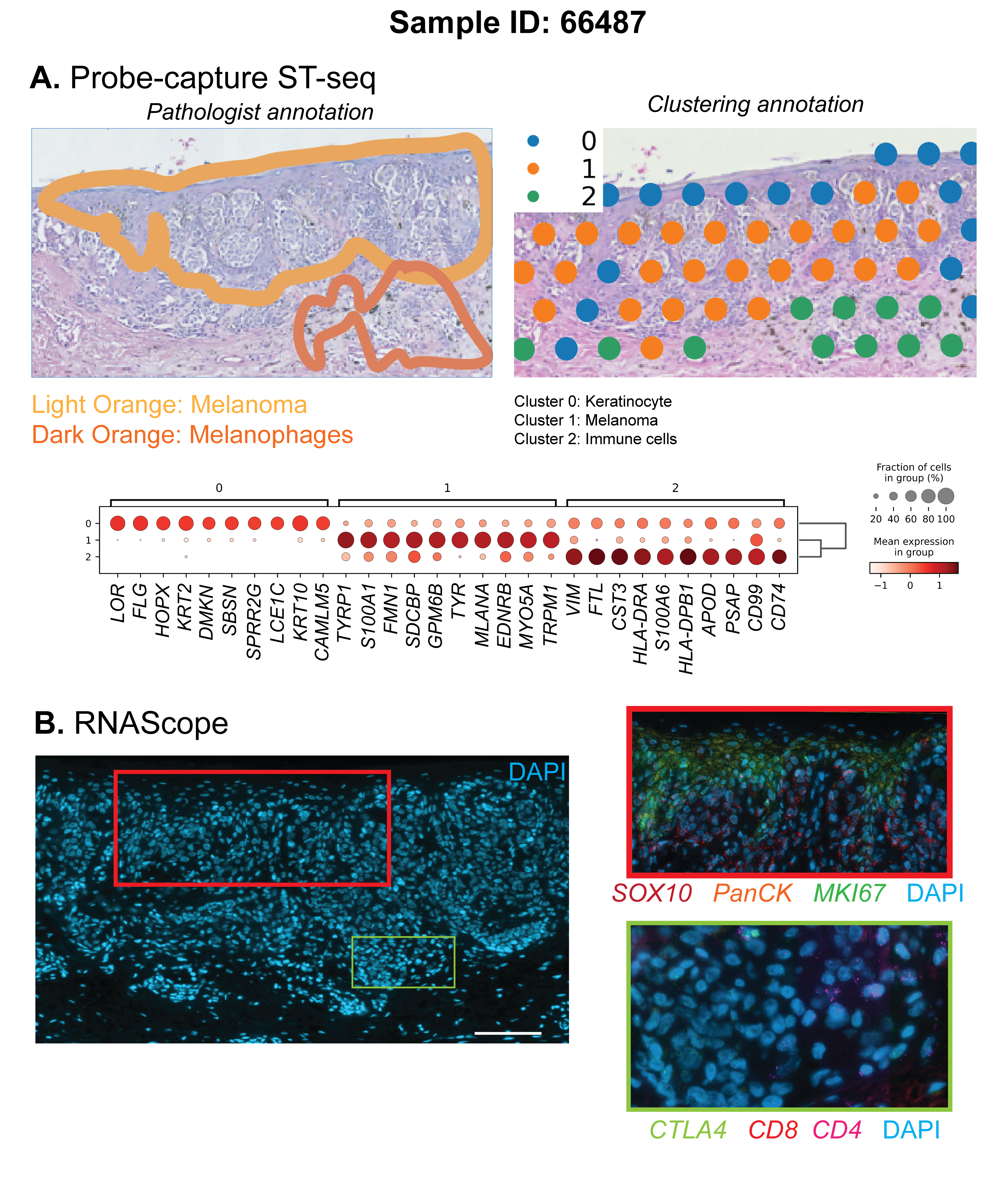


**Figure S6. Comparison of pathologist annotation, Visium clustering, and RNAScope assay.** (A) Visium clustering illustrates the spatial alignment of the annotations with the pathologist annotation. (B) An overview of the section showing nuclei stained with DAPI, including a zoomed-in view of a superficial melanoma region (highlighted by the red line) and an immune cell infiltration area (highlighted by the green line) based on pathological annotation. The melanoma metastasis region near the epithelial layers exhibits expression of cancer markers SOX10, PanCK, and MKI67. Additionally, the immune cell infiltration area shows expression of CD4 T cell markers (CD4, CTL4A) and CD8 T cell markers. The Unsharp Mask filter was applied to enhance image sharpness.

**Figure S7. Comparative analysis of cell type detection using Visium and protein profiling with CODEX**. (A) Annotation of 14 cell types was achieved through deconvolution. A comprehensive scRNA-seq reference was utilised for cell type deconvolution. (B) Comparison with a pathologist's annotation revealed a high proportion of melanocytes within naevus cell nests. Monocytes, dendritic cells (DC), and CD4+ T-cells were identified in lymphocyte-rich regions, while endothelial cells, B-cells, chondrocytes, and fibroblasts were localised to vascular areas. (C,D) Identification of a multitude of cell types and accurate maps of their spatial organisation in tissue sections. Protein profiling via CODEX identified many cell types, especially immune cells such as T cells (by CD8, CD45, CD3e, and CD4), macrophages (HLA-DR, CD163, and CD68), and B cells (by CD19, CD20, and CD21). Other cell types like tumour cells (marked by PMEL), epithelial cells (by PanCK and Keratin 14), and vascular structures (by CD31 and CD34) were also detected. Key immune checkpoints, such as PD-1, PD-L1, IDO1, and LAG3, were also evaluated. However, we noted that observations included significant noise in the signals of PMEL, PanCK, KRT14, and CD163, whereas HLA-DR, and CD4 exhibited auto-fluorescence artifacts. In contrast, Ki67, CD21, CD19, and LAG3 showed a complete absence of staining. Commentary information between ST and CODEX shows unprecedented ability to map cell types.


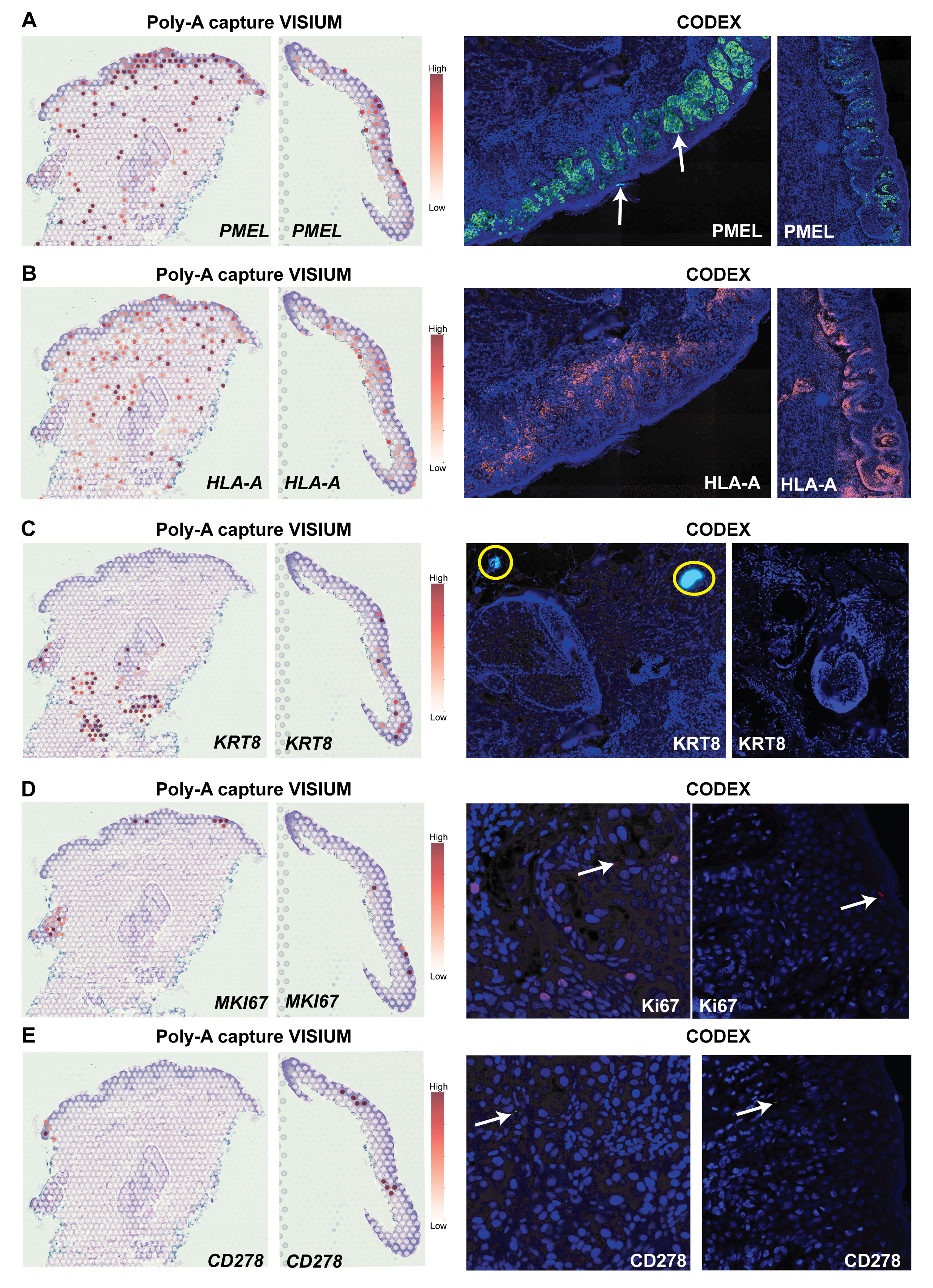


**Figure S8. Comparison of gene detection using Visium and protein detection using CODEX.** On the left, gene expression plotting from Visium is shown for two distinct tissues. On the right, fluorescence images of two different tissues obtained through CODEX are displayed. (A,B) Abundant genes/proteins (PMEL/HLA-A) were effectively detected by both techniques. The noise in the signals detected by CODEX is visibly apparent. C–E) The detection of lowly expressed genes/proteins by both techniques. However, a noticeable discrepancy is observed in the detection sensitivity between CODEX and Visium. Additionally, the presence of autofluorescence artifacts in immunofluorescence images is indicated by the yellow circles.
