# Supplementary material for: Assessing spatial sequencing and imaging approaches to capture the molecular and pathological heterogeneity of archived cancer tissues
Source: J Pathol. 2025 Jan 23;265(3):274–88. doi: 10.1002/path.6383 (PMC11794982; doi:10.1002/path.6383)
Supplement: Supplementary file 2 — Table S1. Information for sample tissues Table S2. Pre‐oligonucleotide‐conjugated antibodies and complementary reporters Table S3. Significantly differentially expressed uTARs in keratinocytes of dysplastic naevus and 10 uTARs in keratinocytes of melanoma Table S4. Top 100 genes per cluster in dysplastic naevus from poly(A)‐capture data – related to Figure 5B Table S5. Top 100 genes per cluster in dysplastic naevus from probe‐capture data – related to Figure 5G Table S6. Top 100 genes per subcluster in dysplastic naevus from poly(A)‐capture data – related to Figure 5D Table S7. Top 100 genes per cluster in melanoma ID 48974 – related to Figure 6B Table S8. Top 100 genes per cluster in melanoma ID9561 – related to Figure 6E Table S9. Top 100 genes per cluster in melanoma ID 15051 – related to Figure 6H [file PATH-265-274-s002.docx]

**Assessing spatial sequencing and imaging approaches to capture the molecular and pathological heterogeneity of archived cancer tissues**

T Vo *et al. J Pathol* <https://doi.org/10.1002/path.6383>

**Supplementary Tables S1–S9**

| **No.** | **Sample ID** | **Sex** | **Age at Diagnosis** | **Year of FFPE processing** | **RIN** | **DV200 (%)** | **Biopsy description** | **Visium method** |  |
| --- | --- | --- | --- | --- | --- | --- | --- | --- | --- |
| 1 | Dysplastic neavus 54013 | M | 25 | 2018 | 2.2 | 47 | Dysplastic naevi, mild-moderate | Poly(A)-Capture |  |
|  |  |  |  |  |  |  |  | Probe-Capture |  |
| 2 | Melanoma 34960 | F | 36 | 2018 | 2.2 | 54 | Malignant, melanoma, breslow 0.48mm | Poly(A)-Capture |  |
|  |  |  |  |  |  |  |  | Probe-Capture |  |
| 3 | Melanoma 66487 | F | 53 | 2018 | 2.2 | 63 | Melanoma arise from dysplastic compound naevus, clark level 2 | Probe-Capture |  |
|  |  |  |  |  |  |  |  |  |  |
| 4 | Melanoma 48974 | F | 79 | 2018 | 2.2 | 76 | Melanoma, clark level 2 | Probe-Capture |  |
|  |  |  |  |  |  |  |  |  |  |
| 5 | Melanoma 9561 | M | 72 | 2008 | 2.2 | 39 | Malignant, Melanoma, Breslow 0.25mm | Probe-Capture |  |
| 6 | Melanoma 15051 | M | 73 | 2008 | 2.5 | 23 | Superficial, Spreading, Melanoma, Breslow 0.3mm | Probe-Capture |  |

**Table S1**. Information for sample tissues.

**Table S2.** Pre-oligonucleotide-conjugated-antibodies and complementary reporters.

| Antibody | Clone no. | Reporter |
| --- | --- | --- |
| CD11b | RM290 | RX030-Cy5 |
| CD11c | AKYP0051 | RX024-Cy5 |
| CD14 | AKYP0079 | RX037-AF750 |
| CD163 | EPR19518 | RX016-AF647 |
| CD19 | RM332 | RX028-ATTO550 |
| CD20 | AKYP0049 | RX007-AF750 |
| CD21 | AKYP0061 | RX032-ATTO550 |
| CD278(ICOS) | AKYP0090 | RX054-ATTO550 |
| CD31 | AKYP0047 | RX001-AF750 |
| CD34 | AKYP0088 | RX025-AF750 |
| CD3e | AKYP0062 | RX045-Cy5 |
| CD4 | AKYP0048 | RX003-Cy5 |
| CD44 | AKYP0073 | RX005-ATTO550 |
| CD45 | AKYP0074 | RX021-AF750 |
| CD45RO | AKYP0059 | RX017-ATTO550 |
| CD57 | HNK-1 | RX049-AF647 |
| CD68 | AKYP0050 | RX015-Cy5 |
| CD8 | AKYP0028 | RX026-ATTO550 |
| CollagenIV | AKYP0083 | RX042-AF647 |
| Foxp3 | AKYP0101 | RX031-AF647 |
| GranzymeB | AKYP0086 | RX041-ATTO550 |
| HLA-A | AKYP0078 | RX004-AF750 |
| HLA-DR | AKYP0063 | RX033-AF750 |
| IDO1 | AKYP0084 | RX027-AF750 |
| Keratin 14 | AKYP0064 | RX002-ATTO550 |
| Keratin 8/18 | AKYP0112 | RX081-AF750 |
| Ki67 | AKYP0052 | RX047-ATTO550 |
| LAG3 | D2G4O | RX055-ATTO550 |
| Pan-Cytokeratin | AKYP0053 | RX019-AF750 |
| PAX5* | RM331 | RX052-ATTO550 |
| PD1 | AKYP0070 | RX046-AF647 |
| PD-L1 | AKYP0097 | RX043-AF647 |
| PMEL* | HMB-45 | RX085-ATTO550 |
| Podoplanin | AKYP007 | RX023-ATTO550 |
| S100A4* | S100A4 | RX064-AF750 |
| SMA | AKYP0081 | RX013-AF750 |
| Vimentin | AKYP0082 | RX022-AF750 |

* Custom Conjugated Antibodies

**Table S3**. Significantly differentially expressed uTARs in the keratinocytes of dysplastic nevus, and ten uTARs in the keratinocytes of melanoma.

| **Sample** | **Putative lncRNA detected** | **Public database ID** |
| --- | --- | --- |
| Melanoma | chr8_102728449_102728749_+_71_0 | HSALNG0067455, HSALNG0067462 (LncBook)  Lnc-ODF1-7 (LNCipedia) |
|  | chr11_75574999_75575199_+_15_0 | Novel |
|  | chr12_118440799_118440949_+_12_0 | Novel |
|  | chr10_121058499_121058749_-_23_0 | NONHSAG007048.2 (NONCODE) HSALNG0081130 (LncBook) Lnc-FGFR2-2 (LNCipedia) CATG00000001057.1\|MICT00000049676.1 (FANTOM-CAT) |
|  | chr1_152665449_152665699_+_45_0 | HSALNG0007237 (LncBook) ENSG00000176075.6/CATG00000079027.1 (FANTOM-CAT) |
|  | chr2_237106299_237106599_+_637_0 | Novel |
|  | chr1_158996799_158996999_-_85_0 | HSALNG0007748 (LncBook) Lnc-AIM2-3 (LNCipedia) |
|  | chr14_105078299_105078749_-_72_0 | HSALNG0104187 (LncBook) |
|  | chr11_78081149_78081349_+_47_0 | HSALNG0085960 (LncBook) |
|  | chr2_159923899_159924149_-_115_0 | Novel |
| Dysplastic Nevus | chr12_52638499_52641099_-_9293_0 | Novel |
|  | chr6_29890799_29891399_+_1267_0 | NONHSAG043383.2 (NONCODE) HSALNG0049094 (LncBook) Lnc-HLA-A-3 (LNCipedia) |
|  | chr20_4573049_4573249_-_690_0 | NONHSAG031179.2 (NONCODE) HSALNG0128326/HSALNG0146906 (LncBook) Lnc-RASSF2-5 (LNCipedia) |
|  | chr7_39812399_39812649_+_1940_0 | Novel |
|  | chr16_55346699_55346899_+_846_0 | Novel |
|  | chr1_151855949_151856449_+_2338_0 | C2CD4D And THEM5 Antisense RNA 1 (HGNC, NCBI Gene, Ensembl) NONHSAG002940.2 (NONCODE) HSALNG0007192 (LncBook) Lnc-OAZ3-2 (LNCipedia) |
|  | chr7_27456299_27456699_-_1306_0 | NONHSAG047210.2 (NONCODE) HSALNG0056889/HSALNG0058897/HSALNG0056893 (LncBook) Lnc-HIBADH-3 (LNCipedia) |

**Table S4.** Top 100 genes per cluster in Dysplastic Naevus from Poly(A)-Capture data - related to Figure 5B.

|  | Cluster 0 | Cluster 1 | Cluster 2 | Cluster 3 |
| --- | --- | --- | --- | --- |
| 0 | COL1A1 | FADS2 | KRT10 | DCD |
| 1 | COL1A2 | MGST1 | KRT1 | MT-ND1 |
| 2 | DCN | FDPS | KRT5 | MT-CO3 |
| 3 | COL3A1 | ALOX15B | TYRP1 | MT-CO1 |
| 4 | SPARC | ELOVL3 | KRT14 | SCGB1D2 |
| 5 | CXCL14 | FAR2 | DCT | MT-ND4 |
| 6 | SFRP2 | CYB5A | COL17A1 | MT-ND2 |
| 7 | PI16 | AWAT2 | KRT15 | SCGB2A2 |
| 8 | FBLN1 | CALML3 | POSTN | MUCL1 |
| 9 | IGFBP5 | FA2H | B2M | SCGB1B2P |
| 10 | CFD | APOC1 | DSC3 | PIP |
| 11 | CORIN | CHCHD10 | MLANA | MT-ND3 |
| 12 | MMP2 | THRSP | HLA-B | MT-CYB |
| 13 | ANGPTL1 | ELOVL5 | LY6D | SLC12A2 |
| 14 | AEBP1 | GAL | HLA-E | MT-ATP6 |
| 15 | FSTL1 | NSDHL | PMEL | MT-ND5 |
| 16 | MGP | ACAA2 | HLA-A | ATP1A1 |
| 17 | LPAR1 | FADS1 | DEGS1 | MT-CO2 |
| 18 | MATN4 | DGAT2 | CD74 | AZGP1 |
| 19 | GSN | KRT79 | DSP | HIF1A |
| 20 | CCDC80 | AGPAT3 | PTMA | APP |
| 21 | SETD7 | ACOT2 | HLA-DRB1 | GAPDH |
| 22 | POGLUT3 | CIDEA | PKP1 | MTRNR2L12 |
| 23 | COL6A2 | CSTB | HLA-DQB1 | AQP5 |
| 24 | DDR2 | BRI3BP | DMKN | SFRP1 |
| 25 | OGN | NDUFV2 | HLA-DRA | DSTN |
| 26 | HMCN1 | SRD5A1 | APCDD1 | TMEM123 |
| 27 | MFAP5 | ACSBG1 | RPL21 | ATP1B1 |
| 28 | SLIT3 | ME1 | DSG1 | MT-ND4L |
| 29 | AOX1 | RDH11 | VIM | ATP6V1B1 |
| 30 | EMILIN1 | TF | RPLP0 | GLS |
| 31 | THBS2 | HMGCS1 | KRTDAP | ZG16B |
| 32 | CTHRC1 | FASN | ATP1B3 | QRICH1 |
| 33 | LRRC15 | ECH1 | CRIP1 | CA6 |
| 34 | C1QTNF3 | TMEM254 | LYZ | SNRNP70 |
| 35 | CERCAM | APMAP | KLF5 | MUC7 |
| 36 | OLFML2B | CRAT | CD81 | MTRNR2L1 |
| 37 | REV3L | INSIG1 | AQP3 | CCDC47 |
| 38 | CD68 | MSMO1 | IFI6 | ITGB1 |
| 39 | MAMDC2 | PM20D1 | GPNMB | NUCB2 |
| 40 | LEPR | CHI3L1 | HMGB1 | ACTA2 |
| 41 | SLPI | DHCR24 | CAST | CLIC4 |
| 42 | ADA | EBP | PPIA | ITGA6 |
| 43 | PXN | ACSL1 | FLG2 | YIPF4 |
| 44 | EPS8L2 | ELOVL1 | HLA-DPA1 | SDC4 |
| 45 | COL6A1 | HMGCR | RPS12 | UQCRB |
| 46 | MICA | SLC25A6 | GAPDH | MELTF |
| 47 | PCM1 | SDC1 | CD44 | ITPR2 |
| 48 | DPP4 | SOAT1 | CA12 | SNHG14 |
| 49 | MFSD10 | UBIAD1 | GLTP | PYGB |
| 50 | CNTFR | PSAPL1 | HLA-C | TXNIP |
| 51 | CUL4B | BCAT2 | GSTP1 | ATP1B3 |
| 52 | DPT | PRDX2 | RPL23A | SAT1 |
| 53 | PDLIM7 | MUC1 | CENPP | CD59 |
| 54 | ADAMTS2 | ADTRP | HOPX | MT-ATP8 |
| 55 | CD163 | DGAT2L6 | SRSF5 | RPL10A |
| 56 | THY1 | C9orf16 | TUBA1B | MYH11 |
| 57 | CFH | ACADVL | PERP | ACSBG1 |
| 58 | ADAM33 | KRT17 | HSPB1 | SOD2 |
| 59 | CNTN1 | SEC14L6 | RHOA | ACTG2 |
| 60 | IGSF10 | ATP5MC3 | MYL12B | SERINC2 |
| 61 | IFIT1 | PGK1 | RPS3 | PLPP2 |
| 62 | MAP1B | COX7B | SFN | COX5B |
| 63 | TIMP2 | DHCR7 | KRT2 | MYL9 |
| 64 | MEGF6 | C1orf122 | MALAT1 | CALM2 |
| 65 | TNFSF13 | PGRMC2 | QPCT | NDRG2 |
| 66 | FBLN5 | GPT2 | TMSB10 | KRT6B |
| 67 | SSC5D | ACOT1 | TMSB4X | MPRIP |
| 68 | CGNL1 | ATP6V0C | RPS19 | OAZ1 |
| 69 | KHNYN | ABHD5 | DST | CD164 |
| 70 | OCRL | NDUFC2 | TYR | A2M |
| 71 | OSR2 | TMEM45A | SYNE2 | NEAT1 |
| 72 | LOX | RPL27 | RPL38 | HSPG2 |
| 73 | ARNT | TECR | TOP2A | TAGLN |
| 74 | RABGEF1 | GPD1 | PDCD4 | MFAP4 |
| 75 | C14orf132 | HSD11B1 | S100A16 | PAFAH1B2 |
| 76 | TTC14 | PRXL2A | EIF5B | KRT7 |
| 77 | IRX5 | FDFT1 | RPL27A | CD44 |
| 78 | CMTM3 | ELOVL4 | TXNIP | SERBP1 |
| 79 | PODN | CALR | RPL19 | RNASE4 |
| 80 | ABL1 | TXN | SLC38A2 | TFCP2L1 |
| 81 | LPAR6 | TKT | CCL27 | CAB39 |
| 82 | ABI3BP | RTN4 | LGMN | NCALD |
| 83 | PDK4 | ACO1 | CD63 | P4HB |
| 84 | TASOR | RPS26 | FGFR3 | ASAH1 |
| 85 | CP | HIBCH | PLP2 | TIMP3 |
| 86 | FBN1 | ALDH3B2 | NEAT1 | FRZB |
| 87 | ADGRA2 | TM7SF2 | RPL7A | DDX39B |
| 88 | UBQLN2 | RPS5 | CXCL14 | FUS |
| 89 | ARHGEF26 | TRIM29 | MT2A | SESN3 |
| 90 | OLFML3 | COX4I1 | CTSC | RPS25 |
| 91 | MS4A4A | CTSV | MAF | ANXA1 |
| 92 | NR3C1 | TUFM | RPLP2 | MYADM |
| 93 | PRRC1 | PHB2 | RPS4X | PIEZO1 |
| 94 | DCLK1 | COX5A | MORF4L1 | DBI |
| 95 | SMAD4 | TMBIM6 | RPL39 | MAPK1 |
| 96 | ELN | ENO1 | RACK1 | AKR1C1 |
| 97 | LMOD1 | NDUFS5 | RPL11 | ZMPSTE24 |
| 98 | C1S | COX7C | KTN1 | NDRG1 |
| 99 | C1QB | PPDPF | RPS27A | CALU |

**Table S5**. Top 100 genes per cluster in dysplastic naevus from probe-capture data - related to Figure 5G.

|  | Cluster 0 | Cluster 1 | Cluster 2 | Cluster 3 | Cluster 4 | Cluster 5 | Cluster 6 | Cluster 7 | Cluster 8 |
| --- | --- | --- | --- | --- | --- | --- | --- | --- | --- |
| 0 | LOR | FADS2 | CORIN | DCN | KRT5 | ACTB | ATP1B1 | KRTDAP | CST6 |
| 1 | KRT2 | FASN | F13A1 | COL1A2 | KRT14 | TMSB4X | TFCP2L1 | DMKN | CTSB |
| 2 | DCD | FADS1 | CTSK | SPARC | S100A2 | PNRC1 | ATP1A1 | GLTP | IER3 |
| 3 | KRT10 | MGST1 | COL6A2 | COL3A1 | KRT15 | GLUL | PPARGC1A | CSTA | KLK6 |
| 4 | FLG2 | ACSL1 | COL6A1 | FBLN1 | COL17A1 | CLU | GABRP | KRT2 | SPNS2 |
| 5 | KRT14 | ACSBG1 | COL1A2 | COL1A1 | TYRP1 | KCTD12 | APP | SCEL | CARD18 |
| 6 | FLG | KRT79 | MMP2 | CFD | PMEL | SPARCL1 | KRT8 | CALML5 | KLK10 |
| 7 | LCE1C | CYP4F8 | COL1A1 | CCDC80 | MLANA | CD74 | GLS | LYPD3 | ACP7 |
| 8 | KRT1 | PM20D1 | FCGRT | COL6A1 | DST | TXNIP | CGNL1 | CASP14 | SLC15A1 |
| 9 | KRT5 | TMEM91 | IFITM3 | IGFBP5 | GSTP1 | CARD8 | SCGB1D2 | EPPK1 | ATP12A |
| 10 | LCE2C | PMVK | AXIN2 | CLEC3B | SLC24A5 | PECAM1 | ATP6V0A4 | HSPB1 | PRSS8 |
| 11 | KRTDAP | CRAT | FBLN1 | FBN1 | TYR | CCL19 | ATP2A3 | SLURP1 | LCN2 |
| 12 | DMKN | APOC1 | COL3A1 | S100A6 | ALDH3A1 | C3 | SFRP1 | DSC1 | PRSS22 |
| 13 | SBSN | AWAT2 | FBN1 | PI16 | CAPN3 | ITM2A | PYGB | GP1BB | JUNB |
| 14 | HOPX | THRSP | TNXB | CTSK | LAMB4 | NRP1 | PDCD4 | KRT78 | PLA2G2F |
| 15 | LCE1A | DHCR24 | TRIM22 | CXCL14 | HIST1H2AG | IGFBP7 | ATP5F1B | DSG1 | SPINK5 |
| 16 | LCE1B | INSIG1 | TYROBP | FSTL1 | HIST1H3D | PTGDS | SDC4 | SLURP2 | SMPD3 |
| 17 | ASPRV1 | FAR2 | RNASE1 | MMP2 | PCLAF | A2M | TBC1D8 | SBSN | KRT6B |
| 18 | DSG1 | ELOVL5 | SFRP2 | GSN | HIST1H4A | VIM | GOT1 | FABP5 | CTSV |
| 19 | C1orf68 | RTN4 | CST3 | CCN5 | WNT4 | B2M | SCGB2A2 | GAN | SPRR4 |
| 20 | SLURP1 | AADACL3 | CXCL14 | FBLN2 | HIST1H2AI | FXYD6 | HIF1A | CLIC3 | CPA4 |
| 21 | DSC1 | SRD5A1 | CD74 | CTHRC1 | HIST1H1B | TNS2 | TIMP3 | KLK8 | TMPRSS11E |
| 22 | LCE2B | MVD | FSTL1 | COL6A2 | TROAP | MYO15B | CKB | KLF4 | ATP6V1C2 |
| 23 | TYRP1 | CYB5A | FTL | C1R | SYT8 | EFHD2 | MTUS1 | PERP | TMEM40 |
| 24 | LCE1D | FA2H | FTH1 | SFRP2 | APCDD1 | HLA-DRA | SAT1 | CYSRT1 | SPRR1B |
| 25 | CALML5 | FDPS | C1S | CXCL12 | TRPM1 | AHR | SLC25A4 | RAET1E | TMEM45A |
| 26 | KPRP | ELOVL3 | CD276 | MGP | ZWINT | HLA-DPA1 | PPP1R1B | WFDC5 | GJB2 |
| 27 | GLTP | ACAA2 | CCL19 | TIMP2 | HIST1H3G | BCAM | SLC12A2 | HOPX | CREG1 |
| 28 | CDSN | DGAT2 | VIM | FN1 | PRC1 | IFITM3 | ANKRD9 | SDR16C5 | C15orf48 |
| 29 | PERP | DDIT4 | AQP1 | PMP22 | MYO5A | CTSC | IVNS1ABP | BNIPL | CITED2 |
| 30 | COL1A1 | TMBIM6 | HLA-DRA | CD99 | HIST1H3H | FLNA | CRACR2B | GRHL3 | SH3D21 |
| 31 | SPRR5 | FABP7 | PLPP3 | CORIN | SLC7A1 | MXRA7 | SOX9 | TPRG1 | BNIP3L |
| 32 | LCE5A | ACAT2 | NFATC4 | CFH | MT2A | FXYD5 | GABARAPL1 | PKP3 | PLA2G4B |
| 33 | HSPB1 | ACADM | HLA-DPA1 | VIM | HIST2H2AB | PPP1R18 | SLC1A5 | JUP | SERPINA3 |
| 34 | WFDC12 | TMEM97 | CIITA | MFAP4 | TPX2 | HLA-DMB | NDRG2 | PPP1R14C | KRT80 |
| 35 | DSP | GPT | DAB2 | PCOLCE | HELLS | ABCA8 | SOD2 | PPL | WIPI2 |
| 36 | SCGB1D2 | ALDH3B2 | COL5A1 | TNXB | POSTN | MRTFB | MELTF | SDR9C7 | ARRDC4 |
| 37 | CRCT1 | AGPAT3 | FGL2 | AEBP1 | HIST1H2BN | LAPTM5 | DUSP4 | AADACL2 | HK2 |
| 38 | CNFN | ERV3-1 | HLA-DPB1 | S100A4 | TOP2A | HLA-F | TLE5 | POF1B | CSTB |
| 39 | PIP | MSMO1 | C1R | SLIT3 | CDH3 | PLXND1 | ENO1 | SERPINB12 | SPRR2A |
| 40 | CASP14 | PLIN5 | CCN5 | S100A10 | AQP3 | RNASE1 | TLE2 | SERPINB5 | TACSTD2 |
| 41 | SPRR2G | CIDEA | IGFBP4 | HTRA1 | H2AFX | CAVIN3 | RUNX1 | LGALSL | ENDOU |
| 42 | COL1A2 | DHCR7 | LAPTM5 | CST3 | KCNAB2 | ZYX | TRPS1 | MYH14 | HMOX1 |
| 43 | BLMH | SOAT1 | TIMP2 | COL6A3 | COL7A1 | CIITA | SGK1 | CTNNBIP1 | CLTB |
| 44 | LYPD3 | BRI3BP | POSTN | SVEP1 | UBE2C | FGL2 | PDLIM4 | BLMH | DNASE1L2 |
| 45 | FABP5 | GAL | HLA-DMB | GAS1 | PTPRZ1 | NTRK2 | DDR1 | BSPRY | FOXO3 |
| 46 | MUCL1 | APOE | CCDC80 | IGFBP4 | HIST1H1E | NPDC1 | SLC39A8 | TGM3 | CRCT1 |
| 47 | COL3A1 | CHI3L1 | SVEP1 | LUM | PLP2 | QKI | KRT7 | RORA | ERO1A |
| 48 | COL17A1 | FBP1 | S100A4 | FTH1 | NUSAP1 | HSPG2 | PLA2R1 | MAL2 | ZFAND5 |
| 49 | LY6D | PNPLA5 | NBL1 | FTL | HIST1H2BO | HLA-E | KRT18 | IVL | S100A8 |
| 50 | JUP | PDZK1 | BGN | FGL2 | NSG1 | S100A4 | ITPR2 | NIPAL1 | ACPP |
| 51 | SCEL | ADTRP | MAMDC2 | TIMP1 | INCENP | ROCK1 | TNS4 | METRNL | UNC5B |
| 52 | PKP1 | DGAT2L6 | CD99 | CD81 | HIST1H4C | CCN2 | AKR1C1 | EREG | TRPV3 |
| 53 | LCE2A | ACOT1 | COL6A3 | RNASE1 | QPCT | TAGLN | XBP1 | CNFN | GSDMA |
| 54 | CSTA | HSD3B1 | TWIST2 | LAMC1 | CDK1 | TPM2 | AFMID | PTGER3 | FBXO32 |
| 55 | KRT15 | TF | TGFBI | MAP1B | MCM7 | COTL1 | TC2N | NECTIN4 | MXD1 |
| 56 | DCN | CLMP | ADGRA2 | SLPI | POU3F1 | LIMS2 | FBXO44 | THEM5 | EGLN3 |
| 57 | CTNNBIP1 | BCAT2 | SYNE1 | SERPING1 | FGFR3 | HLA-DPB1 | CHCHD10 | CARD14 | EGR2 |
| 58 | KLK5 | UBIAD1 | DPT | PLPP3 | TFAP2B | IFITM2 | CAPN2 | PTGS1 | SHF |
| 59 | SYT8 | ACADVL | MYADM | DPYSL2 | HIST1H2BI | TGFB1 | SH3BGRL2 | TUBA4A | PSAPL1 |
| 60 | DSC3 | PECR | PI16 | C1S | MCM3 | DAB2 | TRIM2 | BPIFC | KLK7 |
| 61 | MMP2 | GPD1 | ABCA1 | LPAR1 | KIT | HLA-DMA | SLC12A7 | ANO8 | USP38 |
| 62 | DEGS1 | HILPDA | MPEG1 | CYBRD1 | CENPU | C8orf33 | ZKSCAN1 | EPHX3 | LIPK |
| 63 | CST6 | ECH1 | SELENOM | LGALS1 | ASS1 | CXCL12 | ITGB8 | KLK11 | BCR |
| 64 | WFDC5 | GPX3 | CMTM3 | TWIST2 | CXCL14 | TCF4 | TPD52L1 | ALOXE3 | JMY |
| 65 | HIST1H1B | MUC1 | KCTD12 | MXRA8 | TP63 | RNF213 | TP53INP2 | IL36RN | SLC31A1 |
| 66 | KLF5 | PRXL2A | DNM1 | IFITM2 | MT1E | MPEG1 | SPIRE1 | SEPTIN5 | ABTB2 |
| 67 | AZGP1 | PPARG | CTSC | FCGRT | NCAPD2 | ZNF217 | SLC26A2 | ADGRF4 | QSOX1 |
| 68 | EEF1B2 | SEC14L6 | THBS2 | IGFBP7 | HIST1H3I | DDX39B | NDFIP1 | ALOX12B | BCL2L1 |
| 69 | NCCRP1 | RDH11 | SPARC | ANXA11 | EMP1 | IL32 | SH3BP4 | LIPN | USP2 |
| 70 | GJA1 | TKT | LRP1 | LRP1 | KPNA2 | SORBS3 | PHF1 | RAB11A | H1F0 |
| 71 | ALOX12B | DHRS9 | TSPAN4 | COL5A2 | HIST1H2BB | TPSB2 | SLC9A3R2 | CSNK2A2 | ATG9B |
| 72 | B2M | PNPLA3 | COL18A1 | SRSF11 | OSTM1 | PLVAP | NCALD | SERPINB8 | PRDM1 |
| 73 | FBLN1 | CLSTN3 | FN1 | DNM1 | HDAC11 | TACC1 | CYSTM1 | SEPTIN8 | SQOR |
| 74 | C19orf33 | TLCD4 | TMSB4X | ZEB2 | HIST1H2AE | AQP1 | SLC25A12 | ARG1 | FOXC1 |
| 75 | AQP3 | FKBP5 | WNT4 | SLC29A1 | CBR1 | MAP3K11 | DCD | KRT10 | CA6 |
| 76 | PMEL | MOGAT1 | IL32 | FKBP9 | HIST1H4H | PTK2 | SEMA3C | KLK9 | WASL |
| 77 | IL36RN | PGRMC1 | LAMA4 | APOD | DEK | MOB3B | PRDX3 | TACSTD2 | CRABP2 |
| 78 | ALDH3A2 | ME1 | CTHRC1 | PAM | LSP1 | WIPF1 | EPB41L4B | KLK7 | YWHAQ |
| 79 | SFRP2 | ZNF117 | FBLN2 | TMSB4X | SLC6A9 | EZH1 | THBS1 | PLEKHN1 | NPC1 |
| 80 | LYZ | G6PD | ANGPTL2 | CAVIN1 | TNFRSF19 | S100A6 | PIP | TMEM99 | ADGRL3 |
| 81 | SPARC | CSTB | B2M | SELENOP | PCDH7 | EID1 | ATL2 | RNF225 | TOB1 |
| 82 | SULT2B1 | ACAA1 | CARD8 | DCD | HIST1H2BG | FCGRT | UQCRB | RDH12 | PLK3 |
| 83 | EVPL | PLIN2 | IFI16 | CD63 | SNCA | TGFBR2 | PALMD | ANXA9 | PPIF |
| 84 | CXCL14 | C1orf122 | COL5A2 | ECM1 | HIST1H3A | ZFP36L2 | AQP5 | LY6G6C | LRP10 |
| 85 | TRIM29 | PLIN4 | FOS | PODN | CYB561A3 | PTMS | SESN3 | BICDL2 | FAXDC2 |
| 86 | SLURP2 | LY6E | TIMP1 | ADIRF | TNNT1 | PHLDB1 | RABEP1 | TMEM86A | GJB3 |
| 87 | KLK11 | ACLY | DCN | LCE1C | PSMB8 | EHBP1 | COQ9 | CLDN1 | ASPRV1 |
| 88 | MAF | GK5 | SOD3 | PRRX1 | CLEC2B | GDI1 | NNT | NCCRP1 | OCLN |
| 89 | LAMTOR4 | HSD11B1 | LCE6A | PDGFRB | DDB2 | FLOT1 | TPM2 | NLRP10 | KLK5 |
| 90 | IGFBP5 | ACO1 | SLIT3 | COL5A1 | MLPH | TRIM47 | ACSL3 | CGN | RASAL1 |
| 91 | KRT80 | EBP | HLA-A | CCL21 | HIST1H2AD | PML | HACD3 | TET3 | C4orf3 |
| 92 | POF1B | NTAN1 | ZEB2 | THBS2 | FERMT1 | ECE1 | PPP1CB | SEMA4D | GRHL1 |
| 93 | DBI | IRX6 | HLA-DMA | MYADM | MFSD12 | MYL9 | HES4 | ABCA12 | PARD6B |
| 94 | CD44 | AACS | LCE5A | CERCAM | HIST1H4B | TBC1D2B | VIPR1 | SULT2B1 | SEMA3D |
| 95 | PSORS1C2 | AKR1A1 | PDGFRB | AHNAK | SNAI2 | SEPTIN2 | CTNNB1 | ZNF750 | RND3 |
| 96 | VIM | OAZ1 | CFH | CCL14 | CBX5 | PPP1R12B | MATN2 | SERPINA12 | CDA |
| 97 | ITM2B | UQCRQ | PODN | TPSB2 | HIST1H4E | MFGE8 | DNAJC10 | ATP13A4 | CRYAB |
| 98 | NSG1 | MECR | S100A6 | ADGRA2 | HIST1H4D | ACTA2 | BHLHE40 | SQLE | S100A9 |
| 99 | ANXA9 | CHP1 | CD14 | FGFR1 | TTYH3 | CCL14 | GOT2 | SH3RF2 | EHF |

**Table S6.** Top 100 genes per sub-cluster in dysplastic naevus from poly(A)-capture data - related to Figure 5D.

|  | Cluster 0 | Cluster 1 | Cluster 2 |
| --- | --- | --- | --- |
| 0 | COL1A2 | PMEL | KRT10 |
| 1 | DCN | KRT5 | KRT1 |
| 2 | CD74 | KRT14 | KRTDAP |
| 3 | GSN | DCT | SFN |
| 4 | COL1A1 | COL17A1 | LYPD3 |
| 5 | HLA-DRB1 | TYRP1 | KRT2 |
| 6 | HLA-DRA | DST | LY6D |
| 7 | COL3A1 | KRT15 | ATP1B3 |
| 8 | RNASE1 | CTSD | RPL9 |
| 9 | CST3 | HMGB1 | DEGS1 |
| 10 | SFRP2 | RPL24 | TRIM29 |
| 11 | COL6A2 | NEAT1 | RPS15A |
| 12 | PSAP | RPS28 | RPL22 |
| 13 | MGP | PERP | PABPC1 |
| 14 | HLA-DQB1 | GSTP1 | JUP |
| 15 | CCDC80 | ACTG1 | RPL37A |
| 16 | VIM | IFI6 | DSC3 |
| 17 | SPARC | RPL30 | RPL8 |
| 18 | FBLN1 | RPL35A | RPL4 |
| 19 | HLA-DPA1 | LGALS7B | RPL7A |
| 20 | CCN5 | EEF1G | PERP |
| 21 | IGFBP7 | TUBA1B | LGALS7B |
| 22 | MMP2 | UBB | RPL36A |
| 23 | FSTL1 | TUBB | RPS24 |
| 24 | COL6A1 | MLANA | DSG1 |
| 25 | MT-ND4 | RPL34 | DMKN |
| 26 | B2M | DSP | SNHG29 |
| 27 | CTSK | PTMA | DSP |
| 28 | MT-ND1 | CD9 | PKP1 |
| 29 | SELENOP | LY6D | RPS4X |
| 30 | CFD | RPS5 | GSTP1 |
| 31 | CXCL14 | ATP5F1B | ATP5F1A |
| 32 | MT-ND2 | BTF3 | HSPB1 |
| 33 | TMSB4X | AD000090.1 | RPS29 |
| 34 | DCD | HSPB1 | RPSA |
| 35 | C1R | RPS2 | S100A11 |
| 36 | RPLP0 | RPL19 | DST |
| 37 | APOD | HLA-C | RPS5 |
| 38 | C1S | RPS15 | RBM3 |
| 39 | MT-ND3 | GRN | RPS7 |
| 40 | MT-CYB | RPL22 | COX6C |
| 41 | CXCL12 | LOR | RPS9 |
| 42 | CD81 | KRT10 | KRT17 |
| 43 | SLC25A3 | S100A2 | NEAT1 |
| 44 | RPL10 | RPS14 | MYL12B |
| 45 | ITM2B | RPS23 | RPS15 |
| 46 | TMSB10 | TRIM29 | RPL23A |
| 47 | RPL15 | OAZ1 | RPS16 |
| 48 | CTSB | AQP3 | CD9 |
| 49 | FTL | RPL14 | BTF3 |
| 50 | GAL | TPT1 | SBSN |
| 51 | CD44 | YWHAZ | NACA |
| 52 | FTH1 | MUCL1 | COX7C |
| 53 | MGST1 | RPL8 | MALAT1 |
| 54 | RPL26 | PKP1 | RPL28 |
| 55 | ANXA2 | RPS25 | KRT5 |
| 56 | IGFBP5 | ATP5F1A | RPL38 |
| 57 | ACTB | FLG2 | PPIA |
| 58 | MT-ATP6 | SLC25A5 | RPS21 |
| 59 | RPS13 | RPSA | RPS14 |
| 60 | TIMP2 | GAPDH | SCGB2A2 |
| 61 | TMEM59 | RPL38 | LOR |
| 62 | CD63 | EEF1A1 | RPL10A |
| 63 | TXNIP | RHOA | AQP3 |
| 64 | LAPTM4A | RACK1 | RPS8 |
| 65 | DDX17 | PKM | AD000090.1 |
| 66 | FASN | RPL10A | TMBIM6 |
| 67 | RPS12 | RPS11 | MUCL1 |
| 68 | RPLP2 | KRT17 | SLC25A5 |
| 69 | RPL7 | LDHA | RPS27 |
| 70 | S100A6 | NACA | RPL6 |
| 71 | RPL12 | SFN | YWHAZ |
| 72 | GPNMB | HSP90AB1 | RPL39 |
| 73 | MT-CO3 | RPL4 | PTMA |
| 74 | HLA-B | APOE | HNRNPA2B1 |
| 75 | RPL3 | HNRNPA1 | RPL37 |
| 76 | RPL23 | RPS8 | GJA1 |
| 77 | IGKC | RPS21 | S100A2 |
| 78 | GNAS | MT-CYB | RPL36 |
| 79 | RPS27A | RPL18 | MSMO1 |
| 80 | RPL13A | APCDD1 | RPL21 |
| 81 | HLA-E | KRT1 | IFI6 |
| 82 | EIF1 | S100A10 | MT-ND5 |
| 83 | RACK1 | RPL5 | CALML3 |
| 84 | FADS2 | MT-ND5 | LGALS3 |
| 85 | RPS20 | MT-CO2 | EIF4G2 |
| 86 | APOE | RPL36A | RPL3 |
| 87 | RPL35 | SDC1 | UBA52 |
| 88 | KRT6B | RPL35 | RPL11 |
| 89 | RPS17 | RPS19 | TUBB |
| 90 | LAMP1 | RPS18 | RPS3A |
| 91 | LGALS3 | ACSL1 | NDUFA4 |
| 92 | MT-CO1 | ACTB | TUBA1B |
| 93 | AHNAK | H3F3A | EIF4H |
| 94 | ACSL1 | FADS2 | HNRNPA1 |
| 95 | CD99 | RPL17 | RPL18A |
| 96 | RPL27 | AHNAK | OAZ1 |
| 97 | RPS19 | KRT2 | RPL31 |
| 98 | RPL11 | RPS7 | UBC |
| 99 | APCDD1 | CALML3 | RPL13 |

**Table S7**. Top 100 genes per cluster in melanoma ID 48974 - related to Figure 6B

|  | Cluster 0 | Cluster 1 | Cluster 2 | Cluster 3 | Cluster 4 | Cluster 5 |
| --- | --- | --- | --- | --- | --- | --- |
| 0 | DCN | KRT14 | HLA-DRA | PTPRC | CAVIN1 | PMEL |
| 1 | COL1A2 | TRIM29 | HLA-DPA1 | TRBC2 | PECAM1 | MLANA |
| 2 | FBLN1 | SDC1 | CD74 | TRAC | ACKR1 | BAAT |
| 3 | COL3A1 | CERS3 | LYZ | TMSB4X | CXCL12 | TBC1D7 |
| 4 | COL1A1 | PROM2 | HLA-DPB1 | CXCL9 | CLU | MLPH |
| 5 | CFD | RHOD | C1QB | LTB | EPAS1 | CDK2 |
| 6 | GSN | FXYD3 | HLA-DMB | IL32 | ADIRF | BZW2 |
| 7 | CCDC80 | DSG1 | CTSS | IL7R | IGFBP5 | ANXA5 |
| 8 | APOD | SERPINB5 | HLA-DRB5 | CORO1A | ACTA2 | SLC16A6 |
| 9 | DCD | CLTB | LIPA | TRBC1 | HSPG2 | GPM6A |
| 10 | SPARC | JUP | HLA-DMA | IKZF1 | AQP1 | SLC39A6 |
| 11 | COL6A2 | INAVA | FTL | HLA-F | TNS1 | LGALS3BP |
| 12 | PI16 | DBI | FTH1 | CD3D | LIMS2 | TYRP1 |
| 13 | FSTL1 | CLCA2 | PSAP | TBC1D10C | S100A6 | BACE2 |
| 14 | FBLN2 | DUOX1 | B2M | CD52 | VWF | GPR137B |
| 15 | CCN5 | ESRP1 | TYROBP | CXCR4 | TNS2 | VAT1 |
| 16 | FBN1 | FAM83B | HLA-DRB1 | LAPTM5 | TGFBR2 | CD63 |
| 17 | KRT2 | EVPL | CTSZ | ARHGAP45 | IGFBP4 | TTYH3 |
| 18 | MMP2 | C1orf116 | GZMB | CCL19 | MYL9 | FMN1 |
| 19 | CXCL14 | PPP1R14C | NPL | LIMD2 | A2M | L1CAM |
| 20 | CTSK | DSP | FCER1G | CYTIP | LIFR | PHACTR1 |
| 21 | CLEC3B | KRT5 | TMSB4X | UBD | C3 | MFSD12 |
| 22 | PODN | IRF6 | FPR3 | CDC42SE2 | CD34 | CTNNB1 |
| 23 | TNXB | ALDH3B2 | HLA-DQA1 | SEPTIN1 | PLVAP | SCD |
| 24 | MFAP4 | RAB25 | LAPTM5 | SKAP1 | CFH | QPCT |
| 25 | AEBP1 | SULT2B1 | ADA2 | BIRC3 | TAGLN | TYR |
| 26 | COL6A1 | SPINT2 | CD68 | COTL1 | PLXND1 | SNCA |
| 27 | S100A6 | C19orf33 | PTGDS | RAC2 | C1R | TRPM1 |
| 28 | LOR | GRHL1 | COTL1 | IL2RG | ID1 | EIF4A3 |
| 29 | CYBRD1 | KCTD1 | IGSF6 | WIPF1 | ADCY4 | MIA |
| 30 | KRT10 | LYPD3 | CPVL | CCL5 | TNXB | GSTO1 |
| 31 | ELN | ST14 | SLC15A3 | TCF7 | ENG | SLC7A5 |
| 32 | MGP | IMPA2 | LY96 | SELL | GSN | RAB5B |
| 33 | C1R | CLDN4 | ITGB2 | JAK3 | SELENOM | MAGEC2 |
| 34 | FLG | LAD1 | WDFY4 | CD3E | SLC29A1 | GMPR |
| 35 | SPRR2G | CDH1 | ITGAX | ACAP1 | CCDC80 | HSP90AA1 |
| 36 | KRT1 | S100A16 | LY9 | CD53 | NFE2L1 | MYO10 |
| 37 | LUM | GRHL3 | IGHM | GBP5 | FCGRT | SCARB1 |
| 38 | PDGFRB | KIAA1522 | CTSD | CLEC2D | IFITM2 | TSPAN10 |
| 39 | SLIT3 | SH3RF2 | CIITA | ACTB | FKBP9 | IFI6 |
| 40 | AHNAK | CTNNBIP1 | ST8SIA4 | FYB1 | RNASE1 | KIT |
| 41 | LRP1 | PKP3 | ACP5 | CCR7 | C1S | HSD17B1 |
| 42 | CAVIN1 | IRX3 | CXCR4 | DENND2D | APOD | IGSF3 |
| 43 | CPE | DUOXA1 | JAML | AKNA | STAB1 | RAP1GAP |
| 44 | SOD3 | PDZK1IP1 | SELPLG | TRAJ7 | VIM | SEMA6A |
| 45 | KRTDAP | TMEM45A | AIF1 | TMC8 | MRTFB | GPAT4 |
| 46 | TIMP3 | HSPB1 | IFI30 | TRAJ36 | OLFM1 | SLC24A5 |
| 47 | SFRP2 | CCL27 | MPEG1 | EVL | IL6ST | HMG20B |
| 48 | MRC2 | TMEM40 | IRF8 | IRF1 | MCAM | CAPN3 |
| 49 | SELENOP | DHCR24 | CYBA | STK17B | SERPING1 | BAMBI |
| 50 | PERP | GJA1 | RNF130 | CD3G | SLIT3 | LGALS3 |
| 51 | KRT15 | PKP1 | PARVG | B2M | TPSB2 | WIPI1 |
| 52 | EEF2 | ABCA12 | MAN2B1 | ARHGDIB | TIMP3 | ABR |
| 53 | CFH | TP63 | CHIT1 | PFN1 | TPM2 | ST6GALNAC2 |
| 54 | CXCL12 | NDFIP2 | C1orf162 | TRAJ23 | FLNA | RAB17 |
| 55 | KRT5 | TNS4 | IGKC | LCP1 | KCTD12 | UPP1 |
| 56 | SBSN | ZNF185 | AOAH | CD37 | FBLN2 | PKDCC |
| 57 | IGFBP5 | ZNF750 | LST1 | TRAF3IP3 | RHOB | SHTN1 |
| 58 | GAS1 | TMEM54 | PSTPIP1 | IL10RA | GGT5 | HSPA8 |
| 59 | DSG1 | HIST1H2AC | NAIP | ARHGEF1 | C8orf33 | RAB27A |
| 60 | TPSB2 | IFFO2 | PLA2G7 | PTPN7 | PIK3R1 | TRPV2 |
| 61 | COL12A1 | S100A14 | ACSL5 | VOPP1 | ZFP36L2 | KPNA2 |
| 62 | SERPINF1 | CLDN1 | TRBC2 | TRAJ13 | SVEP1 | PRKD3 |
| 63 | CALML5 | NECTIN4 | SLA2 | CD96 | SPARCL1 | BIRC7 |
| 64 | DMKN | CA12 | LGMN | SPOCK2 | PHLDB2 | GSTP1 |
| 65 | HOPX | CDC42BPG | TNF | CD27 | STMN2 | MCF2L |
| 66 | HTRA1 | DMKN | C1QC | TRAJ5 | MFGE8 | CHMP1B |
| 67 | MEGF6 | GJB3 | ARPC1B | ZAP70 | CXCL14 | LAMA1 |
| 68 | AQP1 | MAL2 | SLA | KLRK1 | IGFBP3 | BRI3 |
| 69 | GAS6 | DEGS2 | CALHM6 | CDC42SE1 | CST3 | ITPKB |
| 70 | C1S | WFDC5 | SYK | CD6 | DPT | EMP3 |
| 71 | KRT14 | PLA2G4F | SRGN | TRAF1 | BCAM | IGFBP7 |
| 72 | NFIB | SERINC2 | CYTIP | TRAJ34 | LTBP3 | C4orf48 |
| 73 | APP | KLK8 | IKZF3 | MMP9 | FSTL1 | CABLES1 |
| 74 | CD99 | KRT80 | CST3 | IDO1 | ZMIZ1 | DNAJA4 |
| 75 | SVEP1 | TMEM134 | CD37 | TNF | RAMP3 | QPRT |
| 76 | THBS2 | SPINT1 | GPR174 | RIPOR2 | ZCCHC24 | RAB32 |
| 77 | CST6 | IL1RN | TIGIT | IKZF3 | CCDC3 | CSTB |
| 78 | FBLN5 | IL20RB | RHOF | TRAJ11 | IFITM3 | RAB7A |
| 79 | COL16A1 | COL7A1 | ITK | LAT | LRP1 | PTTG1IP |
| 80 | ADIRF | CASZ1 | HCST | TRAJ28 | ZFP36L1 | TBC1D16 |
| 81 | DSP | UBC | RNASET2 | TRAJ29 | CYBRD1 | NEDD4L |
| 82 | ABCA8 | RAPGEFL1 | PLD3 | ITK | LAMA4 | ATOX1 |
| 83 | PCOLCE | NECTIN1 | CTSH | CD28 | TMEM173 | CCN3 |
| 84 | PM20D1 | TMEM154 | EVI2A | AGAP2 | NET1 | TIMP2 |
| 85 | LCE1C | MFAP3L | TRAJ6 | TRAJ6 | IGFBP7 | S100B |
| 86 | FLG2 | EHF | CORO1A | FXYD5 | SELENOP | CYB561A3 |
| 87 | FKBP9 | PARD6G | CYBB | LCK | NUCB1 | ACP5 |
| 88 | HSPG2 | DSC3 | C1QA | TAPBP | CALD1 | EMP1 |
| 89 | RNASE1 | PALMD | THEMIS2 | TRAJ17 | FBLN1 | FNDC10 |
| 90 | S100A8 | PERP | ITGA4 | RASAL3 | NRP1 | SDCBP |
| 91 | FADS2 | FAM83H | MS4A7 | DGKA | ID3 | ATP1A1 |
| 92 | CYB5R3 | SERPINB2 | PTPRE | NELL2 | EHD2 | AP2M1 |
| 93 | DPT | KLK11 | IGHG1 | IL2RB | FBN1 | ZNF749 |
| 94 | MUCL1 | BNIPL | BID | RHOH | NPDC1 | NSG1 |
| 95 | AZGP1 | DHCR7 | SNX20 | TRAJ22 | STOM | GPR161 |
| 96 | BGN | DDR1 | FOLR2 | GPR174 | DPYSL2 | LMLN |
| 97 | S100A9 | POU3F1 | PARP15 | CSK | MMP2 | BSG |
| 98 | FN1 | POU2F3 | MPP1 | UBA52 | MGP | ANKRD37 |
| 99 | CD81 | SLC2A1 | BIN2 | BIN2 | FBLN5 | UBB |

**Table S8.** Top 100 genes per cluster in melanoma ID9561 - related to Figure 6E

|  | Cluster 0 | Cluster 1 | Cluster 2 | Cluster 3 |
| --- | --- | --- | --- | --- |
| 0 | PTPRC | COL1A2 | PMEL | KRT10 |
| 1 | TMSB4X | COL1A1 | TYRP1 | KRT1 |
| 2 | RNF213 | COL6A1 | KRT5 | CALML5 |
| 3 | UBA52 | COL3A1 | MFSD12 | DSG1 |
| 4 | IL32 | DCN | NSG1 | LOR |
| 5 | PTGDS | MMP2 | KRT14 | KLK8 |
| 6 | B2M | C1R | S100A1 | KPRP |
| 7 | CCL19 | COL6A2 | HMG20B | KRTDAP |
| 8 | HLA-DRA | FBN1 | IFI6 | FABP5 |
| 9 | HLA-DPA1 | AQP1 | DST | KRT2 |
| 10 | CD74 | FTH1 | CTNNB1 | DMKN |
| 11 | UBC | CD81 | S100A11 | CDSN |
| 12 | HLA-A | SPARC | S100A2 | TRIM29 |
| 13 | LAPTM5 | COL6A3 | GSTP1 | CLTB |
| 14 | TRIM22 | TNC | HSP90AA1 | FLG |
| 15 | VIM | FN1 | HSPB1 | KLK7 |
| 16 | HLA-E | RNASE1 | CAPG | LYPD3 |
| 17 | HIST1H1E | FTL | IFI27 | LGALSL |
| 18 | HLA-C | GSN | SLC2A1 | KLK5 |
| 19 | LYZ | FCGRT | KRT16 | GLTP |
| 20 | HLA-DMA | TYROBP | MT2A | NCCRP1 |
| 21 | ACTR3 | CXCL14 | FXYD3 | CASP14 |
| 22 | HLA-DRB1 | PSAP | EMP1 | SPRR2G |
| 23 | IRF1 | AEBP1 | LY6D | CNFN |
| 24 | ACTB | CST3 | LGALS3BP | S100A7 |
| 25 | HLA-DPB1 | CD74 | EMP3 | JUP |
| 26 | DDX5 | CAVIN1 | AQP3 | GJB2 |
| 27 | C1QC | PTGDS | ATP5F1B | CRCT1 |
| 28 | PFN1 | IGFBP7 | TMED2 | AZGP1 |
| 29 | PCBP2 | IFITM3 | NECTIN1 | CSTA |
| 30 | IGFBP7 | ACTB | RAB25 | PKP1 |
| 31 | CIRBP | SERF2 | COX5B | IVL |
| 32 | C1QB | C1QC | ENO1 | C1orf68 |
| 33 | NOP53 | TYMP | S100A14 | SBSN |
| 34 | HNRNPA3 | HLA-DPB1 | VAT1 | ASPRV1 |
| 35 | TLE5 | SQSTM1 | COX6A1 | S100A14 |
| 36 | PARP14 | LGALS1 | KRT6B | SDC1 |
| 37 | CTSZ | LMNA | CD63 | PKP3 |
| 38 | RNASE1 | TMSB4X | S100A10 | LCE1C |
| 39 | TAPBP | CTSZ | LMNA | CST6 |
| 40 | PLEC | FLNA | GIPC1 | TMEM45A |
| 41 | TAP1 | C1QB | MYC | FLG2 |
| 42 | GNAS | HLA-DPA1 | S100A6 | SLURP1 |
| 43 | STAT1 | MMP14 | C19orf33 | CTNNBIP1 |
| 44 | ARPC1B | HNRNPDL | ANXA2 | RAB25 |
| 45 | TYROBP | VIM | APOE | TUBA4A |
| 46 | WNK1 | MCL1 | GNB1 | HOPX |
| 47 | MYH9 | HLA-DRA | LGALS3 | NECTIN1 |
| 48 | FCGRT | CTSD | TRIM29 | IFFO2 |
| 49 | YPEL3 | HLA-DRB1 | S100A8 | GJA1 |
| 50 | CAVIN1 | CCL19 | MIF | PERP |
| 51 | SRSF5 | ANXA2 | BSG | C19orf33 |
| 52 | FKBP8 | PLXNB2 | DSP | S100A9 |
| 53 | TMEM259 | S100A4 | COX6B1 | LCE2C |
| 54 | HNRNPUL1 | H3F3B | UBB | DSP |
| 55 | HNRNPDL | S100A6 | LAD1 | S100A8 |
| 56 | DCN | PRRC2B | VDAC1 | HSPB1 |
| 57 | FTL | SRSF11 | ECM1 | LY6D |
| 58 | ATN1 | LYZ | BRI3 | PTGS1 |
| 59 | SYNE2 | GABARAP | MICOS10 | LCE1B |
| 60 | AHNAK | CD99 | NCL | LAD1 |
| 61 | FLNA | ZFP36L2 | NME2 | KRT16 |
| 62 | SNRNP70 | UBC | GLUL | AQP3 |
| 63 | MED13L | RSRP1 | PEBP1 | BICD2 |
| 64 | ARPC3 | CTSB | PERP | FXYD3 |
| 65 | PCBP1 | LAPTM5 | CTNND1 | KLF4 |
| 66 | CST3 | AHNAK | GUK1 | DYNLL1 |
| 67 | ARPC5 | TIMP2 | TSPO | KRT14 |
| 68 | ZFP36L1 | BRI3 | RBM3 | SLC2A1 |
| 69 | IFITM2 | ARPC4 | CTSB | KRT6B |
| 70 | C1R | PLEC | SDC1 | GIPC1 |
| 71 | CLIC1 | EIF4A1 | COX7C | S100A2 |
| 72 | PABPC1 | IL32 | PKP3 | ELL2 |
| 73 | HLA-F | DDX5 | S100A7 | TNKS1BP1 |
| 74 | DDX17 | HLA-DMA | FABP5 | NSG1 |
| 75 | TNC | IQGAP1 | KLK8 | METRNL |
| 76 | FTH1 | HNRNPUL1 | SLC25A3 | ECM1 |
| 77 | DRAP1 | HSP90AB1 | PA2G4 | DBI |
| 78 | FBN1 | EEF2 | S100A9 | KRT5 |
| 79 | FLII | S100A10 | EIF4A1 | TSPO |
| 80 | PSAP | IFITM2 | ILF3 | CSTB |
| 81 | HMGB1 | DDX17 | NDUFA13 | TYRP1 |
| 82 | HSPA8 | SRRM2 | CHCHD2 | IFI27 |
| 83 | USF2 | PARP14 | MARCKS | LRP10 |
| 84 | ARHGDIA | SLC2A4RG | BCL2L2 | PPDPF |
| 85 | COL6A2 | HNRNPA2B1 | WARS | LAMP1 |
| 86 | RACK1 | TAGLN2 | GJB2 | RAB5A |
| 87 | DDX39B | NONO | NONO | C4orf3 |
| 88 | EEF2 | GPX4 | RAB5B | AP2S1 |
| 89 | CD99 | CCNI | EIF1 | VAT1 |
| 90 | COL6A3 | QKI | TIMP2 | MYC |
| 91 | RBM3 | RNF213 | SERBP1 | CTNND1 |
| 92 | MACF1 | POLR2L | AP2S1 | PGAM1 |
| 93 | EEF1B2 | KRT2 | EEF2 | ATP5PD |
| 94 | SLC25A3 | MAF | CSTB | RNH1 |
| 95 | GABARAP | ZFP36L1 | TXN | MINK1 |
| 96 | LAMTOR4 | B2M | KRTDAP | RTN4 |
| 97 | CTBP1 | CIRBP | KRT10 | MFSD12 |
| 98 | EDF1 | CD63 | GJA1 | BCL2L2 |
| 99 | FN1 | CD44 | SAT1 | PMEL |

**Table S9.** Top 100 genes per cluster in melanoma ID 15051 - related to Figure 6H

|  | Cluster 0 | Cluster 1 |
| --- | --- | --- |
| 0 | S100A2 | LCE2C |
| 1 | LUC7L3 | LOR |
| 2 | SPARC | FLG |
| 3 | COL3A1 | SPRR2D |
| 4 | FMN1 | SPRR2G |
| 5 | MYO10 | AZGP1 |
| 6 | TYR | CTNNBIP1 |
| 7 | DPP9 | KLK5 |
| 8 | COL6A3 | LCE1C |
| 9 | LRPAP1 | ATP6V1C2 |
| 10 | FAM210B | SPRR5 |
| 11 | HSPB1 | FLG2 |
| 12 | SHTN1 | CST6 |
| 13 | CELSR1 | NCCRP1 |
| 14 | UBR3 | UBC |
| 15 | COL6A1 | GJB3 |
| 16 | POSTN | LCE1B |
| 17 | COL1A2 | KRT23 |
| 18 | FTH1 | CRCT1 |
| 19 | ARF4 | CENPT |
| 20 | SNCA | KRTDAP |
| 21 | GPNMB | LCE5A |
| 22 | ARHGDIA | HOMER2 |
| 23 | CFAP97 | C5orf46 |
| 24 | PTK6 | STUB1 |
| 25 | HTRA1 | CALCOCO1 |
| 26 | FKBP9 | C6orf132 |
| 27 | COL27A1 | CDC34 |
| 28 | SNX5 | SERPINA12 |
| 29 | IFI44 | ATG9B |
| 30 | ERCC1 | EPS8L1 |
| 31 | LTB4R | BSPRY |
| 32 | PCBP1 | PSORS1C2 |
| 33 | TRIP6 | SMPD3 |
| 34 | CD74 | SPRR2B |
| 35 | CHD4 | ASPRV1 |
| 36 | NCL | FOXO3 |
| 37 | COL6A2 | ELOVL7 |
| 38 | MOB3B | XKRX |
| 39 | UBA2 | KLK7 |
| 40 | APP | FURIN |
| 41 | PTGDS | IL36RN |
| 42 | ORC5 | LCE1D |
| 43 | PML | PAQR7 |
| 44 | GRN | LCE3D |
| 45 | DCTD | LCE6A |
| 46 | RAB11FIP3 | MAPKAPK3 |
| 47 | MBP | DUSP14 |
| 48 | ASPH | SPNS2 |
| 49 | PPM1F | ECM1 |
| 50 | TRIM8 | IRAK1 |
| 51 | MID1IP1 | PTGER3 |
| 52 | RANBP2 | TGM1 |
| 53 | RNFT1 | TMEM40 |
| 54 | ACSL3 | SPRR1B |
| 55 | COPA | IGHMBP2 |
| 56 | FTL | RUFY3 |
| 57 | BGN | HPGD |
| 58 | TMEM168 | KDM5B |
| 59 | LPCAT1 | UNG |
| 60 | FOXK1 | TNS1 |
| 61 | MFN1 | C1orf68 |
| 62 | TSPAN14 | GAS6 |
| 63 | ANXA1 | YPEL3 |
| 64 | C1orf52 | LCE2B |
| 65 | CCT5 | KLK8 |
| 66 | MMP14 | BEX3 |
| 67 | PNRC2 | RNF225 |
| 68 | FASTK | SPINK5 |
| 69 | MAPRE1 | ALOX12B |
| 70 | GTF2I | KRT2 |
| 71 | DUSP7 | FCHSD1 |
| 72 | UBQLN4 | LCE3E |
| 73 | SLC2A1 | SLC37A2 |
| 74 | COL1A1 | TGM3 |
| 75 | ITGB4 | RORA |
| 76 | FLNA | TMEM45B |
| 77 | DDX23 | VMP1 |
| 78 | DIDO1 | PPL |
| 79 | WASF2 | LGALS7 |
| 80 | MAF | SPINT1 |
| 81 | EFTUD2 | STAC2 |
| 82 | PARP6 | AKTIP |
| 83 | ALG3 | CUL3 |
| 84 | FAM133B | SLC7A6 |
| 85 | GYS1 | ATG13 |
| 86 | EML4 | PI3 |
| 87 | AP4B1 | ADAP2 |
| 88 | H2AFV | ABHD12B |
| 89 | CDC42BPA | SLURP1 |
| 90 | PTPN1 | GSK3A |
| 91 | LRCH4 | CA6 |
| 92 | RAB1A | YOD1 |
| 93 | DTX4 | RPIA |
| 94 | COL17A1 | PSMD8 |
| 95 | PLXNB1 | H1F0 |
| 96 | XIAP | RAB10 |
| 97 | TBC1D2B | IDS |
| 98 | ARHGEF40 | TTC39B |
| 99 | DNAJA1 | POU2F3 |
